# Predicting Porosity in Grain Compression Experiments Using Random Forest and Metaheuristic Optimization Algorithms

**DOI:** 10.1002/fsn3.70107

**Published:** 2025-03-28

**Authors:** Jiahao Chen, Jiaxin Li, Deqian Zheng, Yan Zhang, Hang Jing, Jianjun Han, Manxing Wang, Runmei Zhao

**Affiliations:** ^1^ College of Civil Engineering Henan University of Technology Zhengzhou China; ^2^ Henan Key Laboratory of Gain Storage Facility and Safety Zhengzhou China; ^3^ Henan International Joint Laboratory of Modern Green Ecological Storage System Zhengzhou China

**Keywords:** bungalow warehouse, compression experiment, grain porosity, metaheuristic optimization algorithm, random forest

## Abstract

Grain stored for long periods is highly susceptible to localized condensation, mold growth, and insect infestations, leading to significant storage losses. These issues are particularly acute in large‐capacity bungalow warehouses, where food security concerns are even more pronounced. The porosity of grain piles is a critical parameter that influences heat and moisture transfer within the grain mass, as well as the ventilation of grain storage. To investigate the distribution pattern of bulk grain pile porosity in bungalow warehouses, this study employs machine learning (ML) techniques to predict grain pile porosity based on compression experiments. Four metaheuristic optimization algorithms—particle swarm optimization (PSO), gray wolf optimizer (GWO), sine cosine algorithm (SCA), and tunicate swarm algorithm (TSA)—were introduced to enhance the random forest (RF) algorithm, and five ML‐based models (RF, PSO‐RF, GWO‐RF, SCA‐RF, and TSA‐RF) for predicting grain porosity were developed. The predictive performance of the five models was analyzed using error analysis, Taylor diagrams, evaluation metrics, and multi‐criteria assessments to identify the optimal ML prediction model. The results indicate that the predictive performance of the four RF‐based hybrid models surpasses that of the single RF model. Among these hybrid models, the TSA‐RF model demonstrated the best predictive performance, achieving *R* ^2^ values of 0.9923 in the training set and 0.9723 in the test set. The TSA‐RF model was employed to conduct a hierarchical prediction of bulk grain pile porosity in the bungalow warehouse. The results indicate that the porosity of the grain pile exhibits a pattern of being higher in the middle and smaller at the edges as the depth of the grain pile increases. The TSA‐RF model developed in this study offers a novel and efficient method for predicting grain porosity, enabling rapid assessments of porosity in bulk grain piles within the bungalow warehouse.

## Graphical abstract

To investigate the distribution pattern of bulk grain pile porosity in bungalow warehouses, this study employs machine learning techniques to predict grain pile porosity based on compression experiments. Five models were developed in this study, with the TSA‐RF model showing the best predictive performance. The TSA‐RF model was employed to conduct a hierarchical prediction of bulk grain pile porosity in the bungalow warehouse.

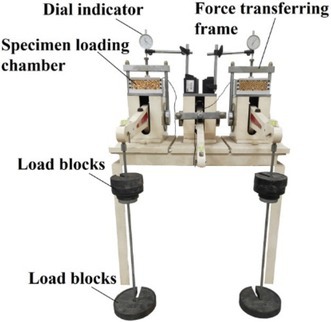

## 1 Introduction

Grain is the fundamental basis for human survival and one of the essential strategic reserve materials for the country. Grain security is crucial for sustaining economic development and social stability. China is a significant grain producer and a major grain storage nation. China's grain reserves are characterized by large warehouse capacity and long storage cycles. During long‐term storage, grains are subject to heat and humidity migration within the pile due to their respiration, microbial metabolism, and the conditions inside and outside the silo. These factors can lead to a significant increase in pests and microorganisms, ultimately compromising the quality and safety of grain storage (Manandhar et al. 2018). Especially in large‐capacity bungalow warehouses, the phenomenon of moisture and heat transfer in grain piles is more pronounced, leading to more serious grain storage issues that are difficult to detect. Large storage capacity, high grain piles, grain gravity, and other factors can lead to compression and deformation of grain, resulting in a non‐uniform distribution of grain pile porosity (Liu et al. 2022). However, grain pile porosity is an important parameter that affects heat transfer and moisture migration within the grain pile (Ge et al. 2023). Therefore, studying the distribution law of grain pile porosity is of great significance for guiding the implementation of grain silo mechanical ventilation, drug fumigation, and other technologies.

The grain pile is composed of two parts: grain seeds and pores, making it a typical porous medium in terms of morphology and structure. The pores of the grain pile are the primary pathways for heat and mass transfer and fluid flow. Therefore, studying the pore structure characteristics in grain heaps is crucial. So far, research on pore structure characteristics has primarily focused on porous media materials such as soil, rock, and coal. However, research on the pore structure of grain piles is relatively limited. Most studies on the pore structure characteristics of porous materials rely on experimental methods and numerical simulations. Gustafson and Hall (1972) determined the porosity of maize using the gas pycnometer method and discovered that the porosity gradually decreased as the weight of the test sample increased. Saki et al. (2020) conducted experiments on 187 rock samples using mercury intrusion porosimetry. They combined the experimental results from the porosimetry with empirical formulas to estimate the permeability of the rocks. Han et al. (2017) utilized gas physisorption analyses to evaluate the pore structure characteristics of shales with varying chemical compositions in the Horn River basin, Canada. Jayas et al. (1995) investigated the relationship between the morphological size of grain particles and porosity, airflow resistance, and explored the mechanism of heat and moisture transfer within the grain pile. The principle of these experiments is to fill the pore structure inside the porous medium with liquid or gas. The pore volume inside the porous material is then derived from the changing volume of the fluid, allowing the porosity of the porous medium to be obtained. However, these methods cannot capture the specific characteristics of the pore structure, and the calculated porosity represents the average porosity of the porous medium.

With the continuous development of science and technology, X‐ray tomography has been gradually applied to study the pore structure of porous media. Neethirajan and Jayas (2008) reconstructed the pore structure of wheat, barley, and flax seeds using X‐ray tomography. Soulaine et al. (2016) characterized the overall pore structure and permeability of rock samples using X‐ray tomography. Markussen et al. (2019) utilized 3D micro X‐ray tomography to analyze the pore structure in siliceous clastic rocks in three dimensions and applied the technique to reservoir rocks in the Edvard Grieg field. X‐ray tomography offers high measurement accuracy; however, it has limitations on the size of the specimen that can be measured, and the equipment cannot determine the porosity of grains under pressure. Therefore, this method is not applicable for determining grain porosity in grain silos. In this study, a custom‐made grain porosity tester was utilized to measure grain porosity under various pressurized conditions. The device accommodates larger grain specimens, thereby preventing porosity measurement inaccuracies caused by smaller specimen sizes.

The method of numerical simulation is also widely used in the study of porous media. It is primarily employed to model and analyze porous media with the assistance of rapidly advancing computer technology. Sobieski et al. (2012) utilized the discrete element method (DEM) to simulate the pore structure of a porous bed and devised a numerical algorithm to construct the pore‐scale flow paths within the simulated spatial structure of the porous bed. Using the DEM to simulate the spatial arrangement of particles in a grain pile, Yue and Zhang (2017) developed a pore‐scale model to predict the resistance to airflow through a grain pile. Lawrence et al. (2013) employed CFD Fluent to simulate the airflow distribution in a grain pile; however, their study overlooked the anisotropy of the particles. Olatunde et al. (2016) modeled the effect of grain structure and porosity on airflow distribution within a grain pile using three‐dimensional computational fluid dynamics.

In recent years, thanks to many breakthroughs in computer science, artificial intelligence and machine learning (ML) techniques have been widely used in various engineering fields (He et al. 2023; Lei et al. 2022; Mahmoodzadeh et al. 2022). Among them, experts in the field of food security storage have also applied ML technology to various aspects of grain storage, successfully addressing numerous practical challenges. In the intelligent ventilation of grain silos, mechanical ventilation is an effective control measure to ensure food security storage. Many experts and scholars are dedicated to researching intelligent ventilation systems for grain silos. Yuzgec et al. (2006) developed a multidimensional nonlinear prediction model using genetic algorithms to predict temperature changes in grain piles during the ventilation of stored grain. By analyzing the data collected in real time, the study demonstrated that the model had a significant impact on regulating the temperature and moisture levels of stored grain. Using meteorological data, Duan et al. (2019) constructed a grain pile temperature prediction model based on the support vector regression algorithm and established a real‐time grain pile temperature monitoring and intelligent ventilation control system. In terms of pest detection in grain storage, the utilization of ML technology enables rapid and precise detection of pests in stored grain piles, ensuring grain safety. Currently, there are various methods for detecting pests in storage grain piles, such as sound detection, electronic nose technology, thermal imaging, ML, deep learning, and other techniques. Shen et al. (2018) developed a detection system for grain storage pests by applying deep neural networks. The results of the study showed that the developed system was able to detect and recognize pests under storage conditions with 88% accuracy. Shi et al. (2020) proposed an improved detection neural network architecture based on R‐FCN to address the issue of detecting and classifying common grain storage insects. The results show that the system is more accurate and faster compared to other detection systems. Eliopoulos et al. (2015) conducted a Hilbert transform on sound signals produced by pests in grain piles and eliminated noise signals from the recorded sounds to deduce potential insect behavioral pulse signals. The results of the study revealed that the monitoring capability of the system improved with the increase in pest density. However, environmental noise had a more significant impact on the monitoring effectiveness of the system. Santiago et al. (2017) preprocessed the sound signals of pests in a grain depot, extracted the feature information from the signals, and classified the pests by constructing an artificial neural network model. The results of the study showed that the accuracy of this pest classification model reached 95.1% and 93.6% in the training set and test set, respectively. In the field of stored grain quality detection, there are factors such as volatility and multiple coupling characteristics that make it challenging to accurately predict the quality condition of stored grain piles. In recent years, due to the continuous development of ML, many scholars have introduced ML techniques into the detection of stored grain quality. Jia et al. (2022) proposed a hyperspectral classification model for corn seed mildew with an accuracy of 92%, which offers technical support and innovative ideas for detecting early mildew disease in corn seeds and for selecting and breeding corn seeds. Wu et al. (2018) proposed a method for the rapid assessment of corn seed quality based on image analysis and support vector machine, achieving an identification accuracy of 97.44%. A significant amount of research has been conducted on ML for intelligent ventilation, pest detection, and quality assessment in grain storage. However, there is limited research on the application of ML for predicting grain porosity.

While traditional methods, such as experimental and numerical simulations, for predicting grain porosity are time‐consuming and labor‐intensive, ML techniques possess a powerful ability to address nonlinear, uncertain, complex, and multivariate problems. Unlike traditional methods, ML models are primarily data‐driven and are not limited by predefined assumptions (Raja et al. 2023). Furthermore, some researchers have integrated ML techniques with simulation experiments, enabling ML models to autonomously learn and reduce the reliance of simulation models on capturing underlying physical or systematic processes (Tongal and Booij 2018; Wang et al. 2022; Yaseen 2021; Zhou et al. 2023). This integration enhances accuracy while significantly reducing the simulation time that would otherwise be required. Consequently, ML models can substantially decrease both the time and cost of research while improving accuracy. ML techniques are gaining popularity in the engineering field due to their significant advantages and robust learning capabilities (Lu et al. 2021; Meng et al. 2024, 2019; Salehi and Burgueño 2018; Sun et al. 2021; Tanveer et al. 2025). Among these techniques, Random Forest (RF) models are particularly favored for their robustness, inclusiveness, and strong ability to mitigate overfitting. RF demonstrates strong performance in both regression and classification tasks, exhibiting a higher tolerance for noise and outliers. In RF modeling, selecting the appropriate combination of hyperparameters is essential for enhancing the model's generalizability, reducing the risk of overfitting, and ultimately improving prediction accuracy (Breiman 2001; Zhou et al. 2023). Common optimization methods include grid search, stochastic search, and metaheuristic algorithms. However, both network and randomized search have their limitations; grid search methods tend to have slow search speeds, while randomized search may be constrained by spatial distribution. As engineering problems grow increasingly complex, the size of the search space and the volume of the models also expand. Consequently, traditional search methods often fall short in meeting the demands for efficient and effective problem‐solving. Metaheuristic optimization algorithms are preferred due to their high efficiency in addressing high‐dimensional complex problems. Therefore, this study employs a metaheuristic optimization algorithm to identify the optimal hyperparameter combinations for RF models.

The primary contribution of this study is the development of novel RF‐based hybrid metaheuristic algorithms for predicting grain porosity, specifically applied to the estimation of bulk grain pile porosity in a bungalow warehouse. To achieve this objective, compression experiments were conducted to gather experimental data on grain pile porosity and its influencing factors, which serve as a database for ML applications. Four robust and applicable metaheuristic optimization algorithms (PSO, GWO, SCA, and TSA) were employed to identify the optimal hyperparameters of the RF model and to predict grain pile porosity. The evaluation of the five models (RF, PSO‐RF, GWO‐RF, SCA‐RF, and TSA‐RF) was performed through error analysis, Taylor diagrams, evaluation metrics, and multi‐criteria assessments, leading to the selection of the optimal grain porosity prediction model (TSA‐RF model). Ultimately, the TSA‐RF model was integrated with the pressure field of the bulk grain pile in a bungalow warehouse to predict the porosity of the bulk grain pile and to derive the distribution pattern of porosity within the bungalow warehouse grain pile. Compared to traditional experimental and numerical simulation techniques, the ML approach utilized in this study offers a novel and efficient method for accurately predicting the distribution pattern of bulk grain pile porosity. This advancement can provide a foundation for the development of hygrothermal regulation and loss reduction technologies for stored grain piles.

## 2 Experimental Measurement of Grain Porosity

### 2.1 Materials

Wheat, corn, soybeans, and paddy used in this experiment were harvested in 2021. Broken grains, immature seeds, and impurities were filtered out using a circular sieve. For each of the different types of grains, 100 grain particles were randomly selected, and their triaxial dimensions were measured using digital vernier calipers. The average length, width, and thickness of various grains were calculated. Equivalent diameters and sphericity of the four grains were calculated using equations (1) and (2), respectively, following the method proposed by Ghodki et al. (2019).

(1) $$D_{p}=\sqrt[{3}]{L\times W\times T}$$

(2) $$S_{p}=\frac{\sqrt[{3}]{L\times W\times T}}{L}$$

where *D*_*p*_ is equivalent diameter; *S*_*p*_ is sphericity; *L* is grain length; *W* is grain width; and *T* is grain thickness.

Grain porosity is calculated as follows:

(3) $$n=1-\frac{\rho_{p}}{\rho_{b}}$$

where *n* is the porosity of the grain; *ρ*_*p*_ is the grain density; and *ρ*_*b*_ is the bulk density.

The initial moisture content of the four grains was obtained by drying them at 103°C for 72 h. The physical parameters of the various grains are presented in Table 1.

**TABLE 1:** Physical parameters of different grains.

Parameters	Symbol	Wheat	Corn	Soybean	Paddy
Grain length	*L*, mm	5.904	11.649	6.272	8.905
Grain width	*W*, mm	3.408	7.422	6.086	2.627
Grain thickness	*T*, mm	3.070	4.170	5.740	1.588
Equivalent diameter	*D*_*p*_, mm	4.230	6.816	5.903	3.327
Sphericity	*S*_*p*_	0.710	0.612	0.981	0.356
Grain density	*ρ*_*s*_, kg/m^3^	1250	1300	1300	1140
Bulk density	*ρ*_0_, kg/m^3^	798	800	716	600
Porosity	*n*	0.425	0.449	0.497	0.521
Moisture content	MC, %	11.05	11.70	9.57	9.83

*Note:* Initial bulk density *ρ*_0_ is the density of the grain pile (cell) when it is not under vertical load; particle density *ρ*_*s*_ is the ratio of the mass of a single grain particle to its volume.

To investigate the influence of moisture content and grain type on the porosity of a grain pile, the same method used by Coşkun et al. (2006) to regulate the moisture content of grains was employed. Distilled water was sprayed on the surface of wheat, maize, soybean, and rice grains, respectively. The samples were then sealed in polyethylene bags and placed in a constant temperature and humidity chamber at 4°C and 50% humidity for 48 h to allow the moisture to equilibrate. Five different moisture contents were prepared for each type of grain, and the results for the four grain moisture contents are presented in Table 2. The average moisture content of multiple grain particles was used to determine the overall moisture content of the grains.

**TABLE 2:** Moisture content of various grain samples.

Grain type	Initial moisture content MC_0_/%	Actual moisture content after formulation MC/%
Wheat	11.05	11.05
		14.96
		15.78
		16.71
		18.38
Corn	11.70	11.70
		14.06
		16.78
		18.39
		21.78
Soybean	9.57	9.57
		12.04
		14.57
		15.20
		18.13
Paddy	9.83	9.83
		14.42
		16.34
		18.82
		23.41

### 2.2 Experimental Apparatus

**FIGURE 1:**
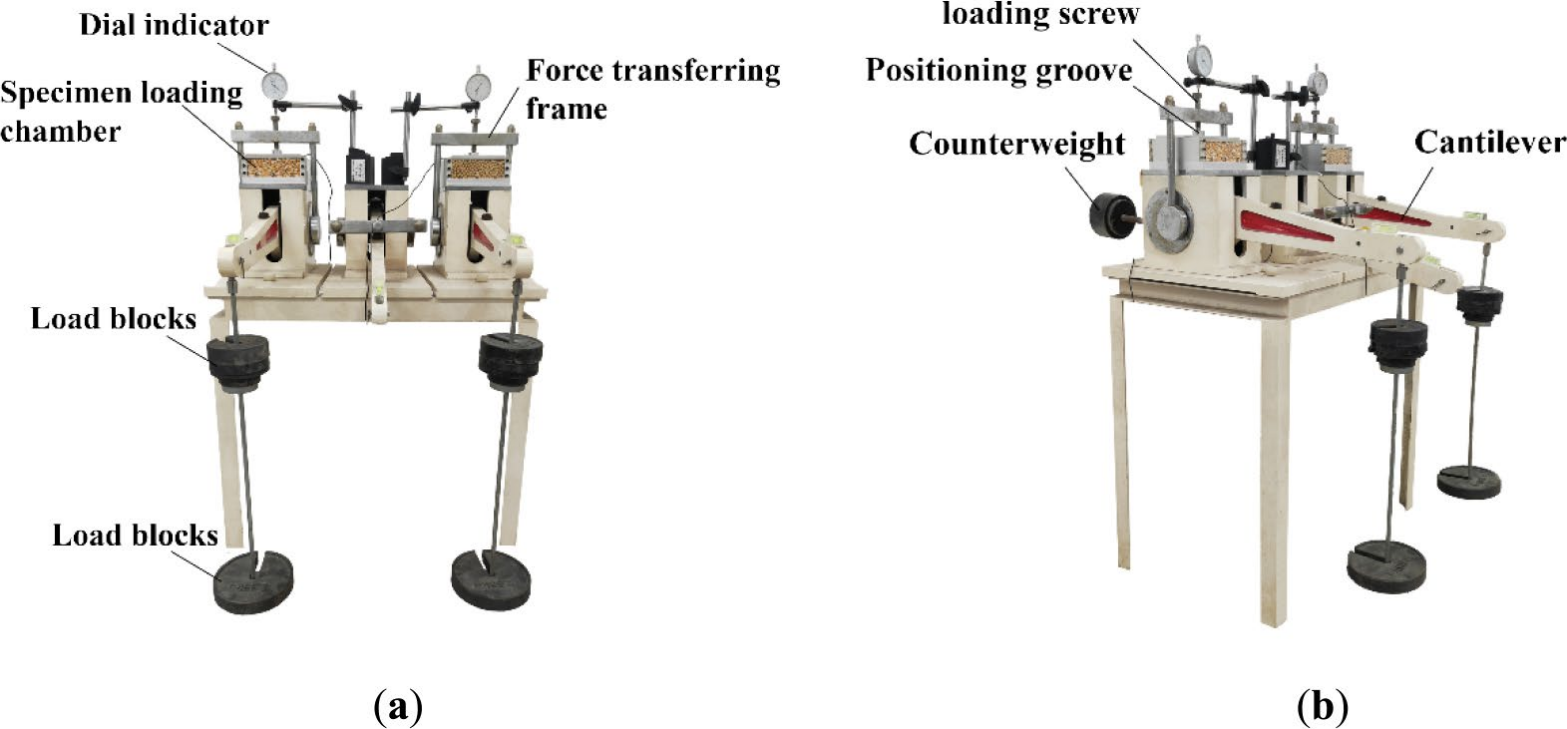
Structure schematic diagram of grain porosity tester (a) Front view, and (b) Side view.

The device used for the compression experiments was a grain porosity tester, as shown in Figure 1. The device is an enhancement of the geotechnical consolidator, capable of applying a vertical pressure of 1200 kPa to assess the porosity of a bulk grain pile under varying vertical pressures. The specimen loading chamber of the grain porosity tester is made of aluminum alloy and a transparent plexiglass plate, and the inner diameter of the box measures 120 mm × 120 mm. In order to enable the loading system to be properly applied to the upper part of the specimen loading chamber, the height of the specimen loading chamber is set to 50 mm. A layer of petroleum jelly was applied to the wall of the specimen loading chamber before the start of the experiment to minimize the friction between the grain seeds and the side wall of the loading chamber during compression. At the top of the loading chamber, a positioning groove is provided for placing the loading screw during the assembly of the apparatus. This ensures uniform pressure on the grain in the specimen loading chamber. A pressure sensor is positioned at the bottom of the specimen loading chamber to measure the vertical pressure on the grain pile.

### 2.3 Experimental Principle

The grain porosity tester in this study can determine the porosity values of each grain type under different pressure states. Due to the relatively hard solid particles of the grain, it is assumed that only the inter‐particle pore space changes during grain compression.

The formula for the initial porosity of grains in their natural stacked state is:

(4) $$n_{0}=\frac{e_{0}}{1+e_{0}}\times 100\%$$

(5) $$e_{0}=\frac{\rho_{w}G_{s}}{\rho_{d}}-1$$

(6) $$\rho_{d}=\frac{M}{V\left(1+\mathrm{MC}\right)}$$

where *n*_0_ is the initial porosity of the grain; $e_{0}$ is the initial porosity ratio of the grain; $\rho_{w}$ is the density of water, kg/m^3^; *G*_s_ is the specific gravity of the grain; $\rho_{d}$ is the dry density of grain, kg/m^3^; *M* is the mass of the grain unit, kg; *V* is the volume of the grain unit, m^3^; MC is the moisture content of the grain.

The formula for porosity of grain specimens during compression is:

(7) $$n_{i}=1-\frac{h_{0}\left(1-n_{0}\right)}{h_{0}-\Delta h_{i}}$$

where $n_{i}$ is the porosity of the grain specimen after loading the ith level of pressure; *h*_0_ is the initial height of the grain pile in millimeters; and $\Delta h_{i}$ is the change in height of the grain pile after loading the ith level of load in millimeters.

### 2.4 Experimental Procedure

Before the experiment started, a layer of petroleum jelly was applied to the inner wall of the specimen loading chamber. Subsequently, an appropriate amount of grain specimens was loaded into the specimen loading chamber. Then the vertical loading system of the grain porosity tester was utilized to load the specimens at 0 kPa, 2.60 kPa, 5.21 kPa, 10.41 kPa, 20.82 kPa, 41.64 kPa, 83.28 kPa, 124.92 kPa, 166.56 kPa, and 208.20 kPa, totaling 10 levels. Each loading was allowed to stand for half an hour to enable sufficient alteration in the pore space of the grain pile within the specimen loading chamber. Each type of grain was subjected to five groups of experiments with varying water content, resulting in a total of 20 experiments. The percentage meter readings in the grain porosity tester were noted, and the porosity under each level of loading was obtained according to Equation (7).

## 3 Database and Data Presentation

### 3.1 Selection and Analysis of Input and Output Parameters

In this study, a total of 200 sets of experimental data were obtained through experiments. Seven parameters, such as grain type, moisture content, grain length, grain width, grain thickness, internal friction angle, and vertical pressure, were used as input variables, while porosity was used as the output variable for modeling and prediction. Table 3 presents the statistical details of the data samples above. Among them, grain types were represented by integer numbers 1 to 4 for wheat, corn, soybean, and paddy, respectively.

**TABLE 3:** Statistics of data samples used for modeling.

Variable	Symbol	Unit	Type	Min	Max
Grain length	*L*	mm	Input	5.764	12.484
Grain width	*W*	mm	Input	2.382	8.178
Grain thickness	*T*	mm	Input	1.451	6.281
Grain type	GT	—	Input	1	4
Moisture content	MC	%	Input	9.57	23.41
Vertical pressure	VP	kPa	Input	0	208.20
Internal friction angle	IFA	°	Input	27.464	41.908
Porosity	*n*	—	Output	0.371	0.532

**FIGURE 2:**
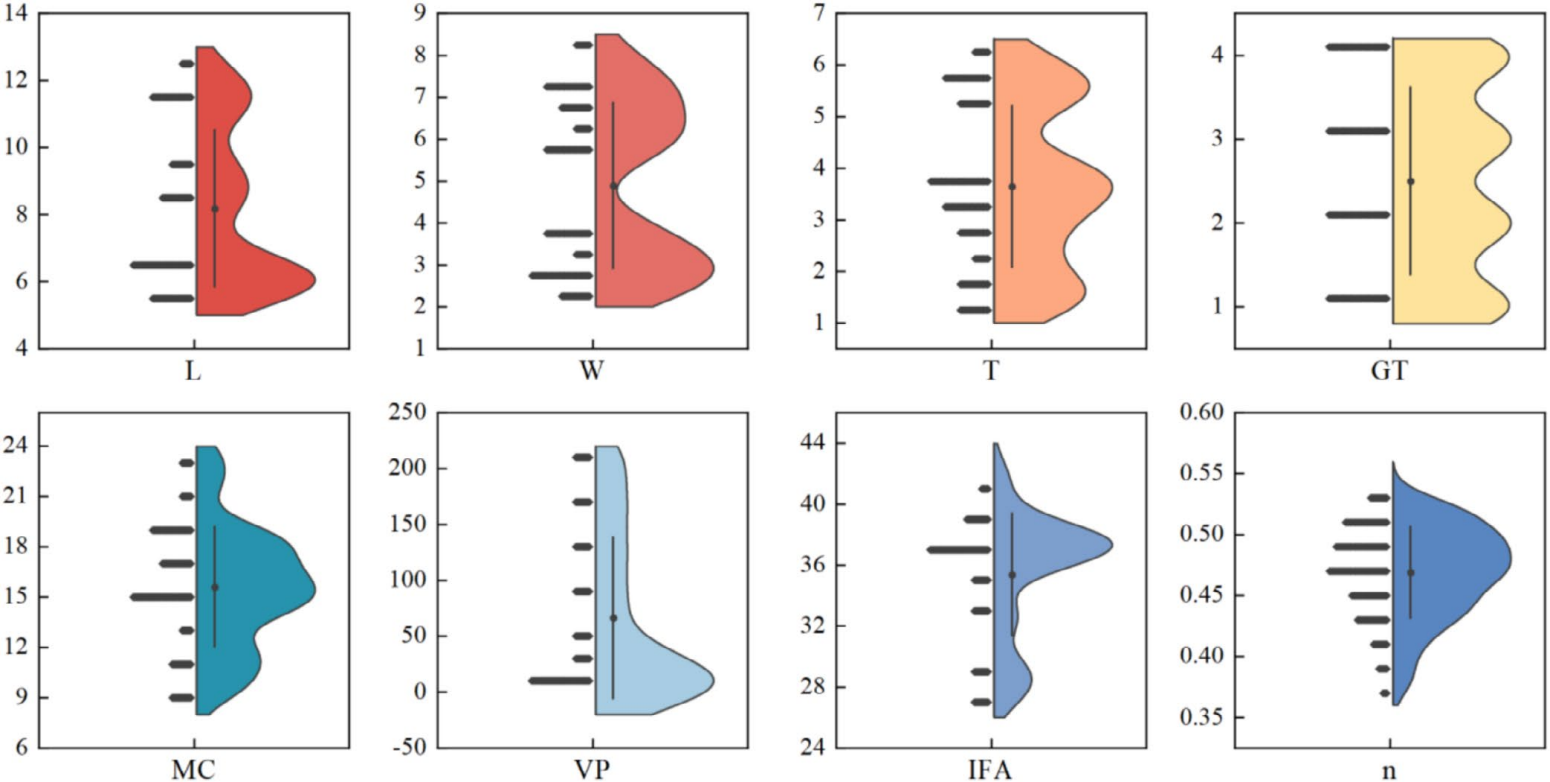
Violin plot of input variables and porosity.

In order to assess the presence of outliers in the database and the correlation between each input variable and porosity, the database was evaluated by creating violin plots and correlation matrix heat maps. A violin plot is a graphical representation of data distribution that displays kernel density estimates to provide more information about shape and distribution (Zaheer et al. 2023). The width of the violin plot represents the data density, while the black dot at the center indicates the median value. Violin plots provide insight into the distribution of multiple data sets and reveal information about skewness and kurtosis. Figure 2 displays violin plots illustrating the distribution of input variables and porosity. The data for each input and output variable in this study are uniformly distributed, with no apparent anomalous data.

### 3.2 Correlation Analysis

In this study, Pearson's correlation coefficient and Spearman's correlation coefficient are chosen as correlation evaluation indices. The correlation coefficient is used to describe the linear correlation between each influencing factor and porosity. By comparison, the influencing factors that are sensitive to porosity can be quickly identified (Sun et al. 2022).

Pearson's correlation coefficient is calculated as:

(8) $$\rho_{X,Y}=\frac{\sum_{i=1}^{m}\left(X_{i}-\bar{X}\right)\left(Y_{i}-\bar{Y}\right)}{\sqrt{\sum_{i=1}^{m}{\left(X_{i}-\bar{X}\right)}^{2}\sum_{i=1}^{m}{\left(Y_{i}-\bar{Y}\right)}^{2}}}$$

where $X_{i}$ represents the variable X, $Y_{i}$ represents the variable Y, $\bar{X}$ represents the mean value of the variable X, $\bar{Y}$ represents the mean value of the variable Y, and *m* represents the total number of sample size sets.

Spearman's correlation coefficient is calculated as follows:

(9) $$\rho_{s}=\frac{\sum_{i=1}^{m}\left(R_{i}-\bar{R}\right)\left(S_{i}-\bar{S}\right)}{\sqrt{\sum_{i=1}^{m}{\left(R_{i}-\bar{R}\right)}^{2}\sum_{i=1}^{m}{\left(S_{i}-\bar{S}\right)}^{2}}}=1-\frac{6\sum d_{i}^{2}}{m\left(m^{2}-1\right)}$$

where $R_{i}$ denotes the rank of the variable X; $S_{i}$ denotes the rank of the variable Y; $\bar{R}$ denotes the average rank of the variable X; $\bar{S}$ denotes the average rank of the variable Y; and $d_{i}$ denotes the difference in rank between two columns of paired variables, $d_{i}=R_{i}-S_{i}$.

**FIGURE 3:**
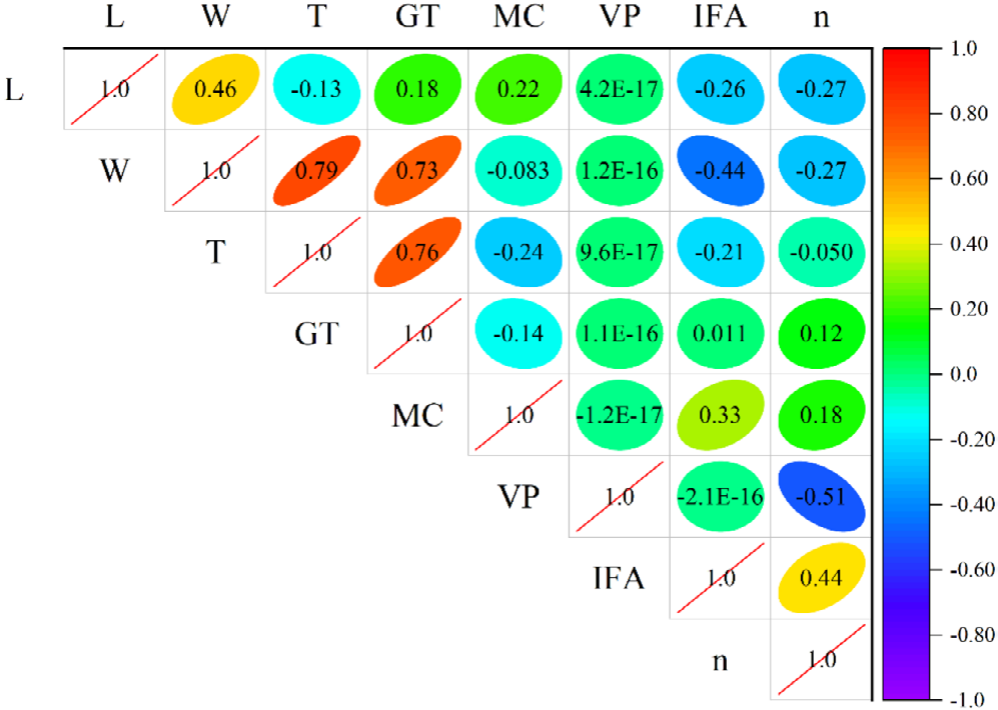
Correlation between parameters based on Pearson algorithm.

**FIGURE 4:**
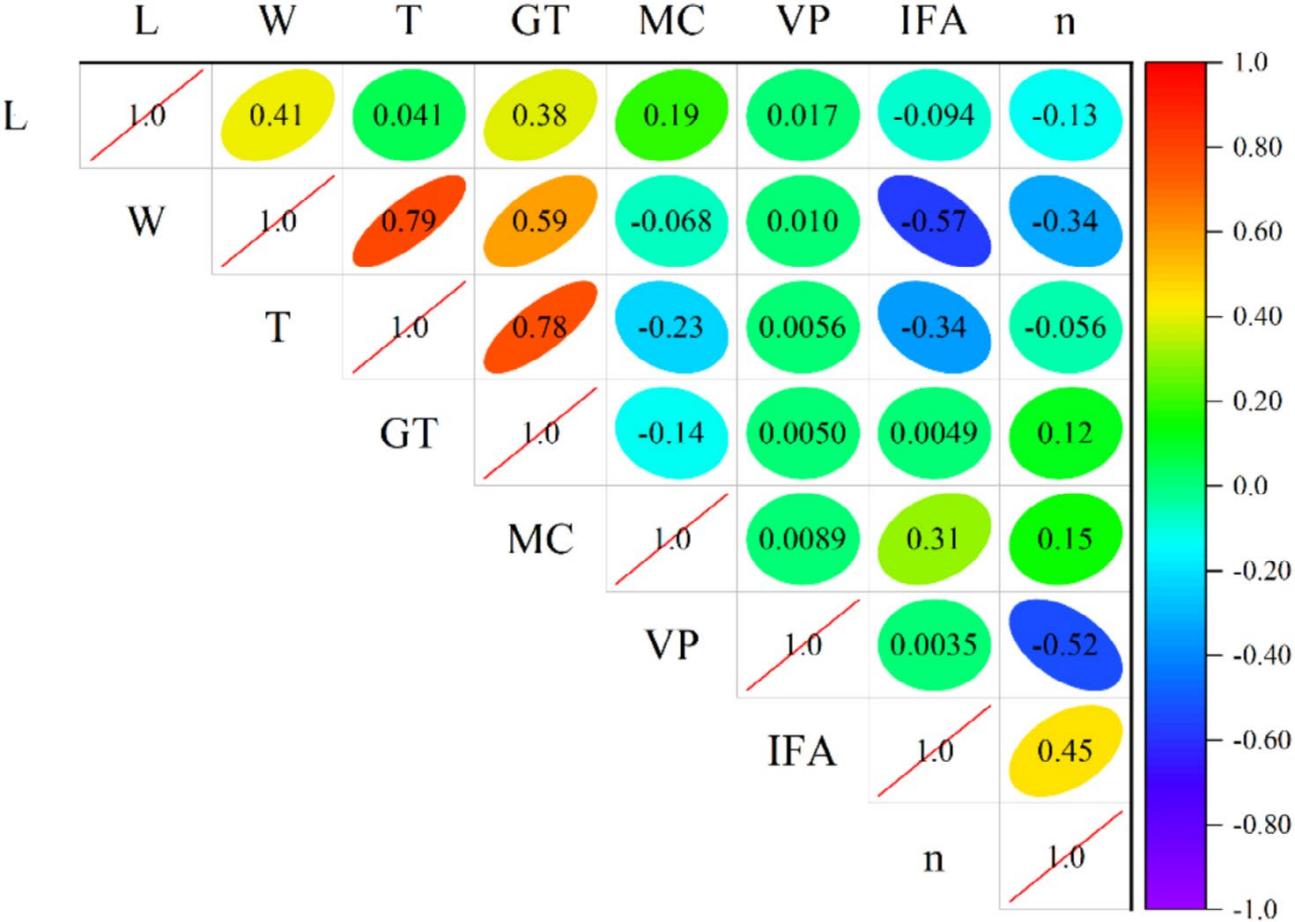
Correlation between parameters based on Spearman algorithm.

The closer the absolute value of the correlation coefficient of two variables is to 1, the stronger the correlation; the closer it is to 0, the weaker the correlation. Figures 3 and 4 utilize a correlation heatmap to display the correlation between data parameters. The values in the plots represent the correlation between two parameters. From Figure 3, it can be seen that the correlation between porosity and vertical pressure (based on Pearson's coefficient) is the strongest, with an absolute value of 0.51, while the correlation between porosity and grain thickness is the weakest, with an absolute value of only 0.05. From Figure 4, it can be seen that the correlation between porosity and vertical pressure (based on Spearman's algorithm) is the strongest, with an absolute value of 0.52, while the correlation between porosity and grain thickness is the weakest, with an absolute value of only 0.056.

### 3.3 Division of the Dataset

The reasonableness and professionalism of dataset partitioning are crucial for model training and effectiveness. A well‐thought‐out division of the dataset is essential for developing a ML model with strong generalization and high robustness (Al Khalifah et al. 2020). In this study, a total of 200 sets of data were obtained. The data were divided into two parts according to a 7:3 ratio, with 140 sets used as the training set and 60 sets used as the test set.

### 3.4 Data Preprocessing

Before establishing the grain porosity prediction model, it is necessary to normalize the raw data to facilitate data extraction and improve the model's accuracy. Subsequently, ML modeling can be conducted based on the normalized data. There are seven influencing factors of grain porosity selected in this study, and the magnitudes of these factors vary from one another. If the original data is directly applied to ML modeling without any processing, the prediction results of the model will be affected to some extent, leading to slower model training efficiency and poorer training results. In order to avoid these phenomena, it is necessary to normalize the original data.

To mitigate the impact of varying scales among variables on the model and to guarantee the accuracy and reliability of the constructed model all the variables in the database were normalized to fall within the range of [0,1], and then the subsequent model training was conducted (Guo et al. 2022). The normalization formula is:

(10) $$x_{n}=\frac{x_{\mathrm{ij}}-\min_{k}\left(x_{\mathrm{kj}}\right)}{\max_{k}\left(x_{\mathrm{kj}}\right)-\min_{k}\left(x_{\mathrm{kj}}\right)}$$

where $x_{n}$ is the normalized result; $x_{\mathrm{ij}}$ is the data to be normalized; $\max_{k}\left(x_{\mathrm{kj}}\right)$ is the maximum value of the data; and $\min_{k}\left(x_{\mathrm{kj}}\right)$ is the minimum value of the data.

## 4 Research Methodology

### 4.1 Particle Swarm Optimization

The particle swarm optimization (PSO) algorithm was proposed by studying the social behavior of birds. In this algorithm, a large number of particles are created and placed in an N‐dimensional search space. The particles continuously search for the optimal position, with each particle representing a potential solution to a problem. In the process of searching for the optimal solution, the particles dynamically adjust their positions and speeds based on their own movements and those of other particles. Eventually, the particle swarm will approach the optimal position based on the fitness function (Hou et al. 2021).

In the PSO algorithm, the mathematical model for updating the positions and velocities of all particles is as follows (Singh and Sharma 2018):

(11) $$x_{\mathrm{new}}=x+v_{\mathrm{new}}$$

(12) $$v_{\mathrm{new}}=\mathrm{\omega v}+c_{1}p_{1}\left(p_{\text{best}}-x\right)+c_{2}p_{2}\left(g_{\text{best}}-x\right)$$

where *p*_1_ and *p*_2_ represent random values between 0 and 1; *c*_1_ and *c*_2_ are optimization parameters; *ω* is the inertia weight coefficient; *x*_new_ and *x* represent the old and new positions, respectively; *v*_new_ and *v* represent the old and new velocity values, respectively; *p*_best_ is the optimal position of the selected particles; and *g*_best_ is the optimal position of all particles. For a detailed description of the PSO algorithm, refer to Kennedy's study (Kennedy and Eberhart 1995).

### 4.2 Gray Wolf Optimizer

**FIGURE 5:**
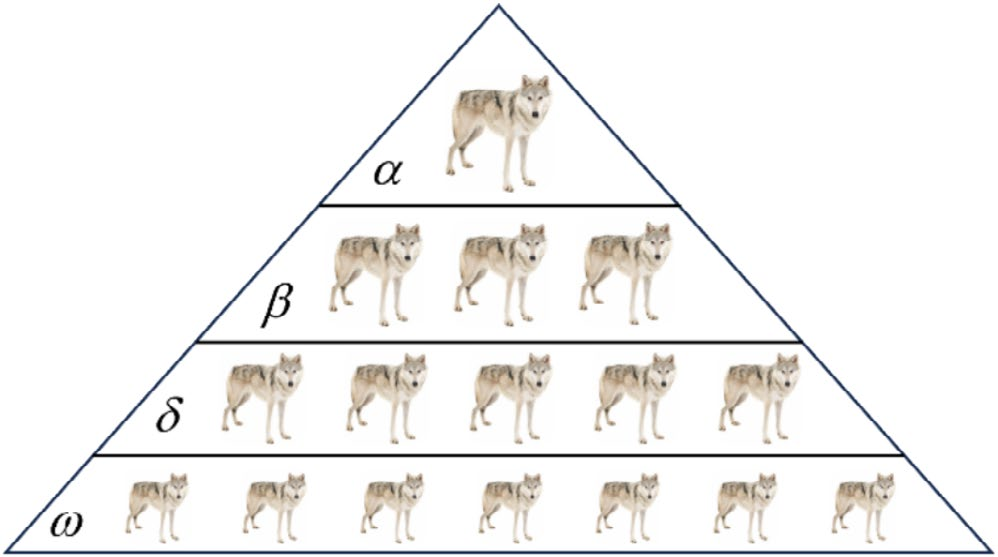
Hierarchy of gray wolves (decreasing dominance from top to bottom).

The gray wolf optimizer (GWO) algorithm is a metaheuristic algorithm based on group intelligence proposed by Mirjalili et al. The algorithm is inspired by the social structure of the gray wolf swarm (Mirjalili et al. 2014). The hierarchy of gray wolves is illustrated in Figure 5, showing four distinct types of gray wolves in their social structure: *α* wolves, *β* wolves, *δ* wolves, and *ω* wolves. Their social status decreases from left to right.

The four ranks of pack wolves represent the four solutions sought by the GWO optimization process, with *α* representing the optimal solution, *β* and *δ* being the two suboptimal solutions, and *ω* being the candidate solution. The mathematical model of the GWO algorithm consists of three main steps.

**FIGURE 6:**
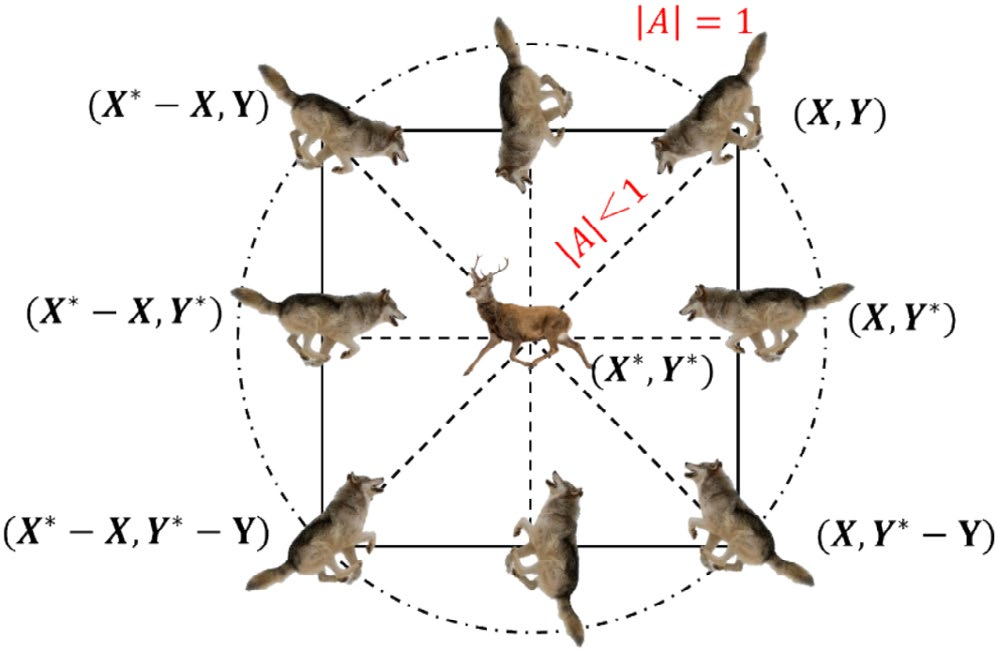
Position vector of wolves.

1. Surround the prey. Gray wolves will lock onto the prey's position and then encircle the prey during hunting. This behavior can be simulated using mathematical models.

(13) $$D=\left|C\cdot X_{p}\left(t\right)-X\left(t\right)\right|$$

(14) $$X\left(t+1\right)=X_{p}\left(t\right)-A\cdot D$$

where *D* is the distance between the gray wolf and the prey; *A* and *C* are coefficient vectors; *t* is the number of iterations; *X*_p_(*t*) and *X*(*t*) are the position vectors of the prey and the gray wolf after *t* iterations, respectively, and *X*(*t* + 1) denotes the position vector of the gray wolf after *t* + 1 iterations, as illustrated in Figure 6.

Where A and C are calculated as:

(15) $$A=2a\cdot r_{1}-a,a=2-\frac{2t}{T_{\max}}$$

(16) $$C=2\cdot r_{2}$$

where *a* is the convergence factor, which linearly decreases from 2 to 0 as the number of iterations increases; $r_{1}$ and $r_{2}$ are random vectors in the range [0,1]; and $T_{\max}$ is the maximum number of iterations.

**FIGURE 7:**
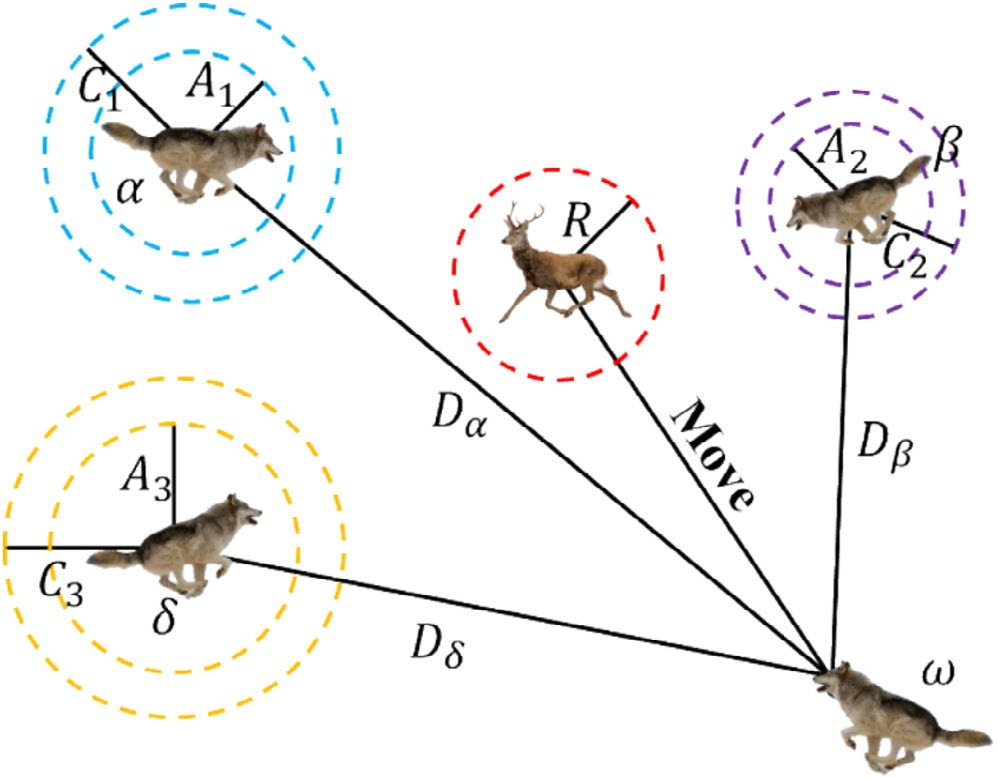
Location update process of gray wolf algorithm.

2 The hunting process. *ω* can update its positions based on the positions of wolves *α*, *β*, and *δ*. For this purpose, Mirjalili et al. (2014) proposed Equations ((17), (18), (19)) for calculating the new positions of wolf *α*, wolf *β*, and wolf *δ*. The position updating process is shown in Figure 7.

(17) $$\left\{\begin{array}{c} D_{\alpha }=\left|C_{1}\cdot X_{\alpha }\left(t\right)-X\left(t\right)\right|\\ D_{\beta }=\left|C_{2}\cdot X_{\beta }\left(t\right)-X\left(t\right)\right|\\ D_{\delta }=\left|C_{3}\cdot X_{\delta }\left(t\right)-X\left(t\right)\right| \end{array}\right.$$

(18) $$\left\{\begin{array}{c} X_{1}=X_{\alpha }\left(t\right)-A_{1}\cdot D_{\alpha }\\ X_{2}=X_{\beta }\left(t\right)-A_{2}\cdot D_{\beta }\\ X_{3}=X_{\delta }\left(t\right)-A_{3}\cdot D_{\delta } \end{array}\right.$$

(19) $$X\left(t+1\right)=\frac{X_{1}+X_{2}+X_{3}}{3}$$

where $D_{\alpha }$, $D_{\beta }$, and $D_{\delta }$ represent the distances between wolves *α*, *β*, and *δ* and other gray wolves, respectively; $A_{1}$, $A_{2}$, $A_{3}$, and $C_{1}$, $C_{2}$, and $C_{3}$ represent the coefficient vectors; $X_{\alpha }\left(t\right)$, $X_{\beta }\left(t\right)$, and $X_{\delta }\left(t\right)$ represent the position vectors of *α*, *β*, and *δ* wolves, respectively, after *t* iterations; and $X_{1}$, $X_{2}$, and $X_{3}$ represent the vectors of the individual gray wolves' movements toward wolves *α*, *β*, and *δ*, respectively.

**FIGURE 8:**
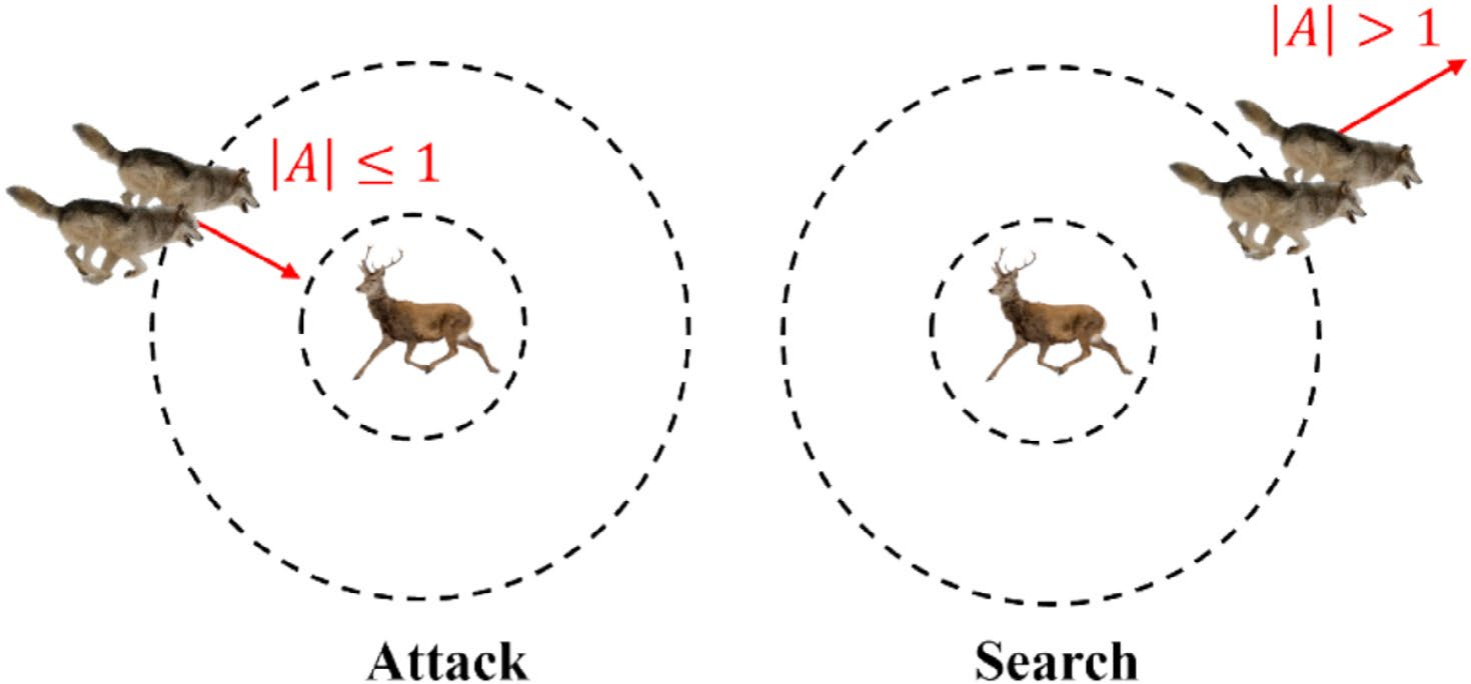
Relationship between the gray wolf and its prey.

3 Attack the prey. The behavior of the wolves is determined by the value of |A| in Equations (14) and (15). When |A| > 1, it indicates that the algorithm is in the global search phase. When |A| ≤ 1, the wolves will launch an attack to capture the target that has been locked. As shown in Figure 8.

### 4.3 Sine Cosine Algorithm

The sine cosine algorithm (SCA) is a new swarm intelligent optimization algorithm proposed by Mirjalili in 2016. It is based on a combination of sine and cosine functions and utilizes multiple stochastic parameters and adaptive variables to fine‐tune the algorithm optimization process (Mirjalili 2016). The SCA algorithm, like other swarm intelligence optimization algorithms, can provide an initial value for each solution based on a priori information. If the a priori information is not available, an initial set can be randomly generated. These solutions are then updated based on their positions using the following equations:

(20) $$x_{i}^{t+1}=\left\{\begin{array}{c} x_{i}^{t}+r_{1}\times \sin\left(r_{2}\right)\times \left|r_{3}p_{i}^{t}-x_{i}^{t}\right|,r_{4}<0.5\\ x_{i}^{t}+r_{1}\times \sin\left(r_{2}\right)\times \left|r_{3}p_{i}^{t}-x_{i}^{t}\right|,r_{4}>0.5 \end{array}\right.$$

where $x_{i}^{t}$ denotes the position of the current solution in dimension *i* at the *t*th iteration; $p_{i}^{t}$ denotes the optimal solution in dimension *i* at the *t*th iteration; and *r*_1_, *r*_2_, *r*_3_, and *r*_4_ are random numbers, where $r_{2}\in \left[0,2\pi \right]$, $r_{3}\in \left[0,2\right]$, and $r_{4}\in \left[0,1\right]$. The parameter *r*_1_ indicates the region (or direction of movement) of the next position, either within the space between the random solution and the optimal solution or outside of it. The parameter *r*_2_ determines the distance moved toward or away from the optimal solution during the iteration. Parameter *r*_3_ is a random weighting factor that enhances (*r*_3_ > 1) or diminishes (*r*_3_ < 1) the effect of the optimal solution in defining the distance. Parameter *r*_4_ controls the switching of the iterative equations for the sine–cosine function. The parameter *r*_1_ adaptively balances global exploration and local exploitation of the algorithm with the following expression:

(21) $$r_{1}=a-t\frac{a}{T}$$

where *t* is the current iteration number; *T* is the maximum iteration number; and *a* is a constant.

**FIGURE 9:**
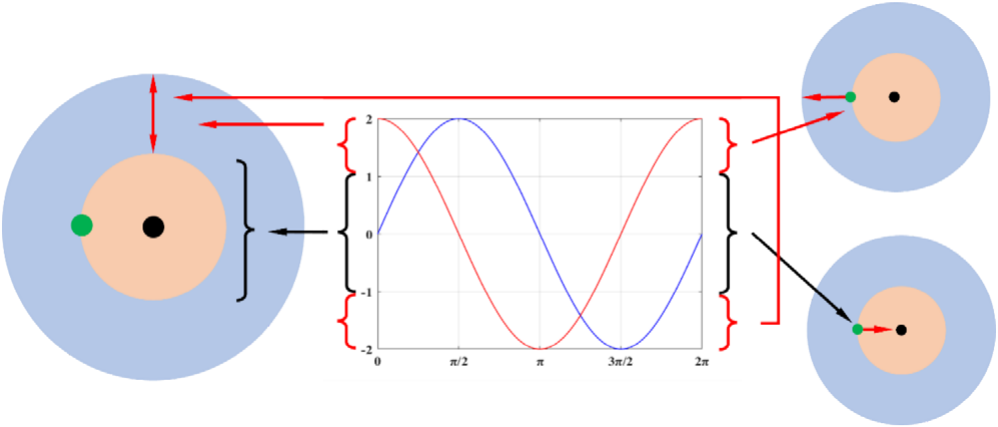
Optimization process of the SCA.

Figure 9 illustrates the process of determining the optimal solution using the SCA. From the figure, it can be seen that the sine and cosine functions oscillate within the interval [−2, 2]. If the value range of the sine or cosine function falls within the interval [−1, 1], the algorithm initiates local exploitation. If the value range of the sine or cosine function is within the interval [−2, −1] or [1, 2], the algorithm initiates a global search in the solution space. Within the specified range, the sine cosine optimization algorithm smoothly transitions from the global exploration phase to the local exploitation phase using both the sine and cosine functions. During the optimization process of the SCA, the candidate solutions update their positions near the current optimal solution and continuously move toward the optimal regional position.

### 4.4 Tunicate Swarm Algorithm

**FIGURE 10:**
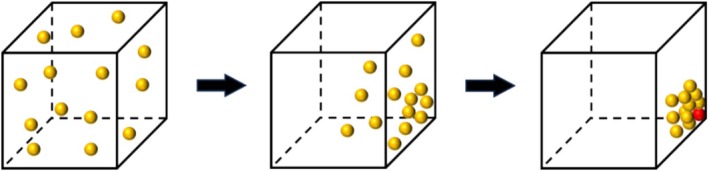
The TSA search strategy.

The tunicate swarm algorithm (TSA) is a metaheuristic algorithm proposed by Kaur et al. The algorithm is a swarm intelligence optimization algorithm inspired by the foraging behavior of tunicates to simulate tunicate jet propulsion and swarm behavior (Kaur et al. 2020). The jet propulsion of tunicates consists of three main components: avoiding conflicts between tunicate individuals, moving in the direction of the optimal tunicate position, and approaching the optimal tunicate position. The TSA search strategy is shown in Figure 10.

1. Avoiding conflicts among tunicates. The new positional formula for calculating tunicates is shown below.

(22) $$A=\frac{G}{S}$$

(23) $$G=c_{2}+c_{3}-F$$

(24) $$F=2\cdot c_{1}$$

where *A* is the new search position; *G* is the gravity force; *F* is the water flow advection in the deep ocean; *c*_1_, *c*_2_, and *c*_3_ are random numbers between the intervals [0,1]; and *S* is the social force among the tunicate individuals, which is computed as follows:

(25) $$S=\left|P_{\min}+c_{1}\cdot \left(P_{\max}-P_{\min}\right)\right|$$

where *P*_min_ and *P*_max_ represent the initial and auxiliary velocities of the social interaction of the tunicate individuals, with values of 1 and 4, respectively.

2 Move toward the optimal tunicate position. After avoiding conflicts between tunicates, each tunicate in the swarm moves toward the optimal tunicate position. The calculation formula is as follows:

(26) $$\mathrm{PD}=\left|\mathrm{FS}-r_{\text{and}}\cdot P_{p}\left(t\right)\right|$$

where *PD* is the distance between the food and the tunicate; *FS* is the position of the food source, which is the optimal position; *r*_*and*_ is a random number within the range of [0, 1]; $P_{p}\left(x\right)$ is the position of the tunicate in the current iteration; and *t* is the number of current iterations.

3 Converge toward the best tunicate. Individuals of the tunicate will gradually move closer to the position of the best tunicate, which is calculated by the following formula:

(27) $$P_{p}\left(t^{\prime }\right)=\left\{\begin{array}{c} \mathrm{FS}+A\cdot \mathrm{PD},\;\text{if}\;r_{\text{and}}\geq 0.5\\ \mathrm{FS}-A\cdot \mathrm{PD},\;\text{if}\;r_{\text{and}}<0.5 \end{array}\right.$$

where $P_{p}\left(t^{\prime }\right)$ is the updated position.

4 Swarm behavior. To simulate the mathematical model of the swarming behavior of tunicate animals, the first two optimal solutions are saved, and the positions of the remaining tunicate individuals are updated according to the best tunicate positions. The mathematical model of the behavior is defined as:

(28) $$P_{p}\left(t+1\right)=\frac{P_{p}\left(t\right)+P_{p}\left(t+1\right)}{2+c_{1}}$$

### 4.5 Random Forest

The random forest (RF) algorithm, proposed by Breiman in 2001, is an ensemble ML method that combines a large number of decision trees. It operates by drawing multiple samples from the original dataset through bootstrap resampling and constructing a decision tree for each bootstrap sample. The final prediction is obtained by aggregating the predictions of all individual trees, typically through a majority voting process (Yu et al. 2020).

**FIGURE 11:**
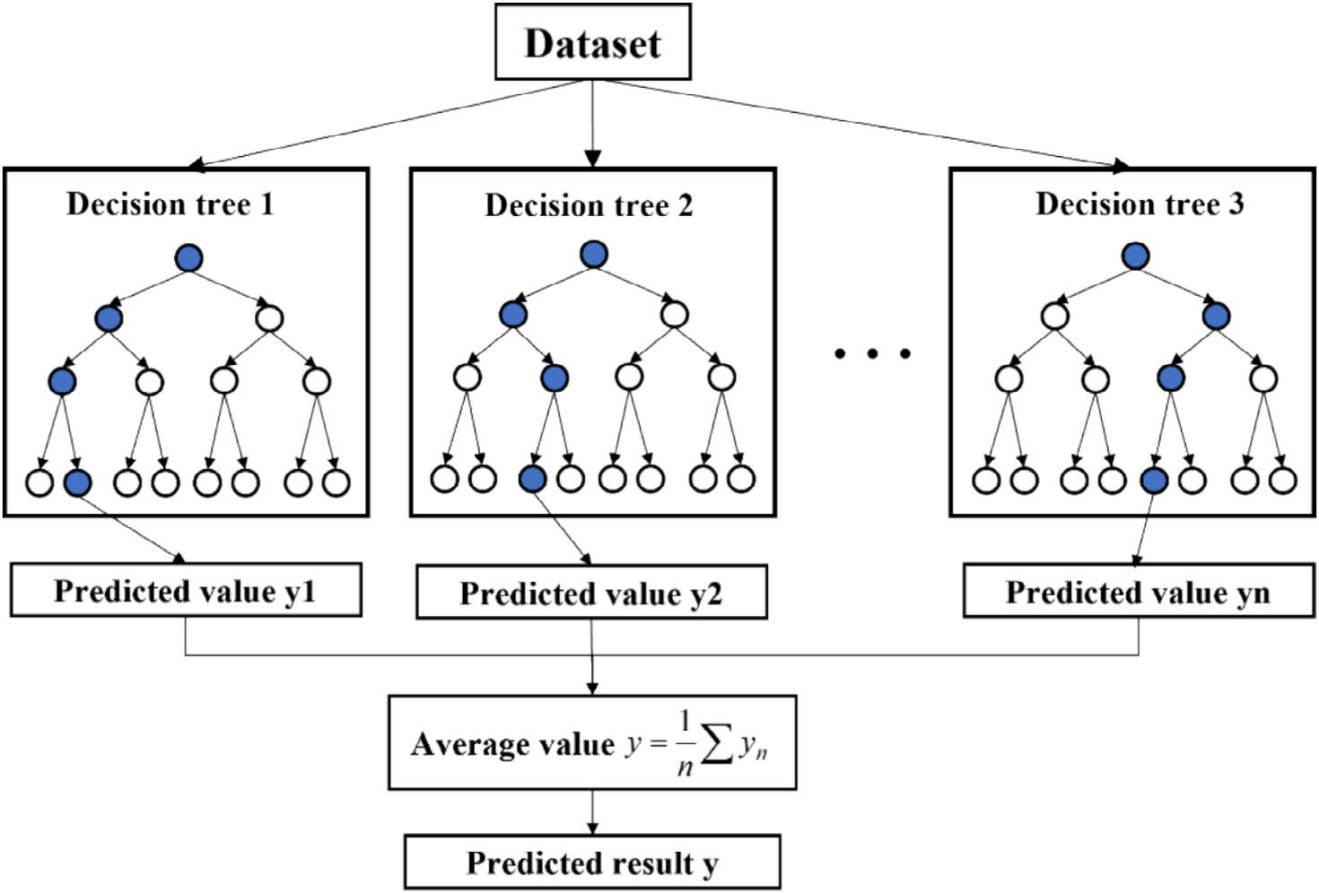
RF algorithm structure schematic.

The RF algorithm is straightforward and easy to implement. It demonstrates strong performance in both regression and classification tasks, exhibits good tolerance for noisy data (such as outliers and missing values), and is less prone to overfitting (He et al. 2023). The structure of the RF algorithm is schematically illustrated in Figure 11. The construction process of the RF model primarily involves the following steps (Han and Xue 2024):

1. Randomly extracting samples from the original dataset and constructing *n tree* subsets of samples using the bagging method.
2. Randomly selecting *mtry* features from the total set of features using the random subspace method for node splitting to construct a decision tree.
3. Calculating the average of the outputs from each decision tree to make the final prediction.

For a detailed description of the RF algorithm, refer to Breiman's study (Breiman 2001). The key hyperparameters of random forests include the number of decision trees (*ntree*) and the number of features considered during node splitting in each tree (*mtry*). The RF algorithm functions as a regression analyzer, while the four metaheuristic algorithms (PSO, GWO, SCA, and TSA) mentioned earlier are employed to optimize the hyperparameters of random forests, thereby constructing hybrid models (PSO‐RF, GWO‐RF, SCA‐RF, and TSA‐RF) based on random forests.

### 4.6 RF‐Based Modeling of Grain Porosity Prediction

Random forest is fundamentally a constraint‐based ensemble learning method. It offers several advantages, including efficient operation that enhances the training speed of samples, high prediction accuracy, and strong performance in handling large volumes of complex data. Additionally, it is less susceptible to overfitting. However, RF has notable shortcomings during execution. For instance, when the dataset contains significant noise, the RF model may still exhibit overfitting. Furthermore, if the input model has numerous feature divisions, it can adversely affect the model's decision‐making, leading to suboptimal fitting performance. To address the various deficiencies present in the traditional RF model when operating independently, four optimization algorithms (PSO, GWO, SCA, and TSA) are introduced to enhance its performance.

**FIGURE 12:**
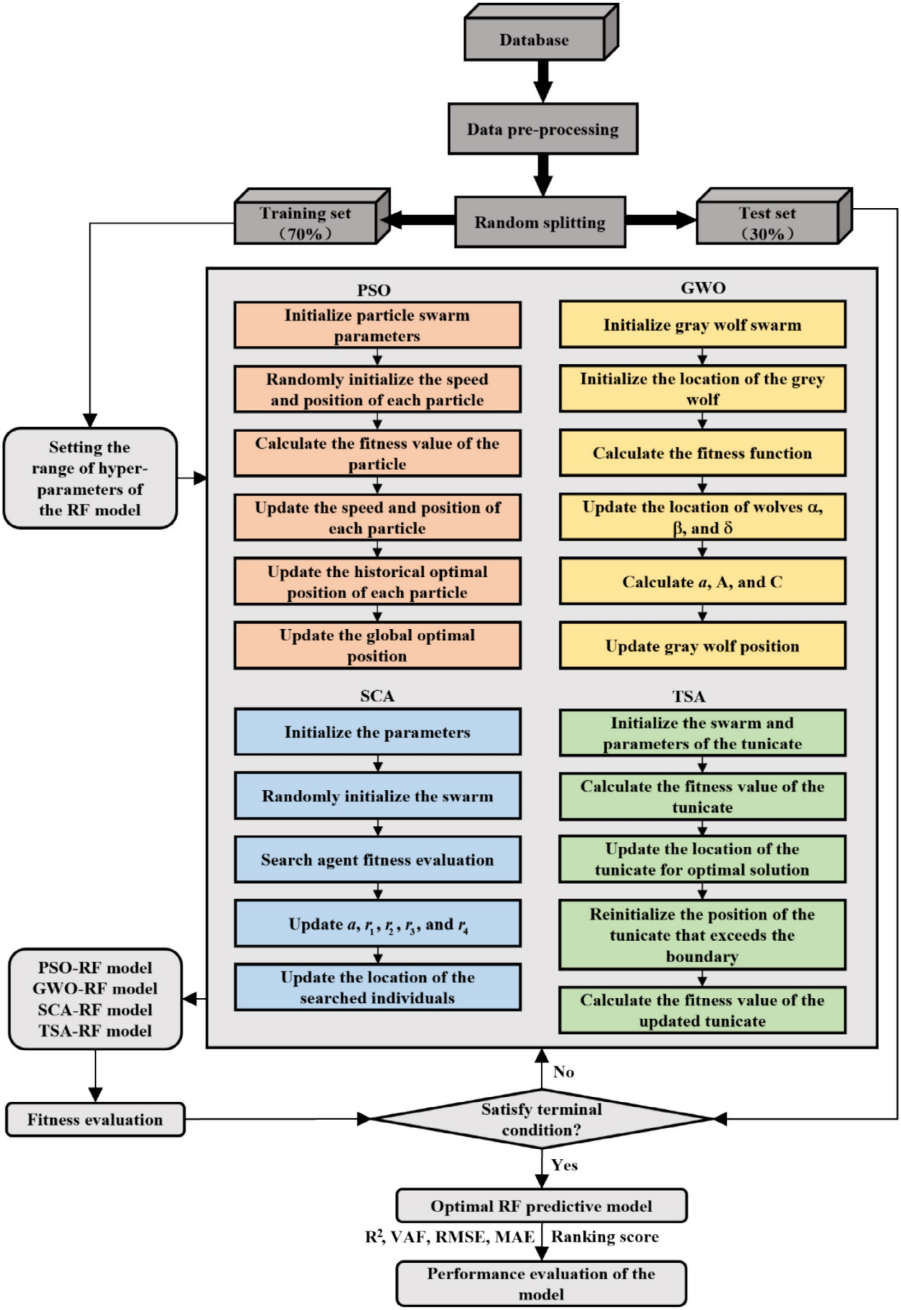
Flowchart of RF‐based models for predicting grain porosity.

The metaheuristic algorithm plays a vital role in the optimization process for identifying the optimal hyperparameters of the RF model, as this significantly enhances the accuracy of the results (Medawela et al. 2023). Figure 12 illustrates the flowchart of the predictive model for grain porosity based on RF regression. The primary steps of the modeling process are as follows:

1. The construction of the database involved normalizing the data before dividing it into a training set and a testing set, with 70% allocated for training and 30% for testing.
2. Set the objective function. The mean square error (MSE) serves as the objective function, and its calculation formula is $\text{Error}=\mathrm{MSE}\left\{Y_{i},\hat{Y_{i}}\right\}$. Where $Y_{i}$ represents the actual value of the sample and $\hat{Y_{i}}$ denotes the predicted value of the model $\hat{Y_{i}}=\mathrm{RF}\left(\text{ntree},\text{mtry}\right)$. The hyperparameters, ntree and mtry, are constrained within the ranges of [1, 600] and [1, 7], respectively, and both hyperparameters must be integers.
3. Initialize the parameters for the optimization algorithm. Set the population size and the number of iterations for the metaheuristic algorithms. The number of iterations is fixed at 150 for each optimization algorithm, while the population sizes are set to 25, 50, 75, and 100, respectively.
4. Calculate the fitness value and record the best fitness value during each iteration to identify the optimal hyperparameters progressively.
5. The optimal hyperparameter combination for the hybrid RF model is achieved when the value of the optimal fitness stabilizes and the maximum number of iterations is reached.

## 5 Results and Analysis

### 5.1 Performance Indices for the Assessment of Models

The purpose of constructing a grain porosity prediction model based on ML is to express the complex nonlinear relationship between grain porosity and its influencing factors and to predict grain porosity using the constructed model. Therefore, some intuitive quantitative data or evaluation criteria are needed to assess the accuracy of grain porosity prediction models or to compare the strengths and weaknesses of various models. This will help improve the performance of grain porosity prediction models.

In this study, four evaluation metrics, namely the coefficient of determination (*R* ^2^), variance accounted for (VAF), root mean square error (RMSE), and mean absolute error (MAE), are selected to assess the model's performance (Koopialipoor et al. 2019; Li et al. 2018). The calculation formula is as follows:

(29) $$R^{2}=1-\frac{\sum_{i=1}^{m}{\left(Y_{i}-\hat{Y_{i}}\right)}^{2}}{\sum_{i=1}^{m}{\left(Y_{i}-\bar{Y_{i}}\right)}^{2}}$$

(30) $$\mathrm{VAF}=\left(1-\frac{\mathrm{var}\left(Y_{i}-\hat{Y_{i}}\right)}{\mathrm{var}\left(Y_{i}\right)}\right)\times 100$$

(31) $$\text{RMSE}=\sqrt{\frac{\sum_{i=1}^{m}{\left(Y_{i}-\hat{Y_{i}}\right)}^{2}}{m}}$$

(32) $$\mathrm{MAE}=\frac{\sum_{i=1}^{m}\left|Y_{i}-\hat{Y_{i}}\right|}{m}$$

where *m* is the total number of samples; *i* is the number of sample sequences; $\bar{Y_{i}}$ is the average of the true value of the samples; $Y_{i}$ is the true value of the samples; and $\hat{Y_{i}}$ is the predicted value of the samples. *R* ^2^ is used to assess the linear fit of the model, and its value ranges from 0 to 1. The closer the *R* ^2^ value is to 1, the better the fit of the model; conversely, the closer the *R* ^2^ value is to 0, the worse the fit of the model. VAF is an indicator used to assess the degree of correlation between the predicted and actual results of a model. The higher the VAF value, the better the predictive performance of the model, and the closer the model predictions are to the actual results. RMSE is the square root of the ratio of the square of the deviation between the predicted value and the true value to the sample size m. MAE is the average of the absolute errors between the predicted value and the actual value. The lower the values of RMSE and MAE, the greater the predictive accuracy of the model.

### 5.2 Model Development

**FIGURE 13:**
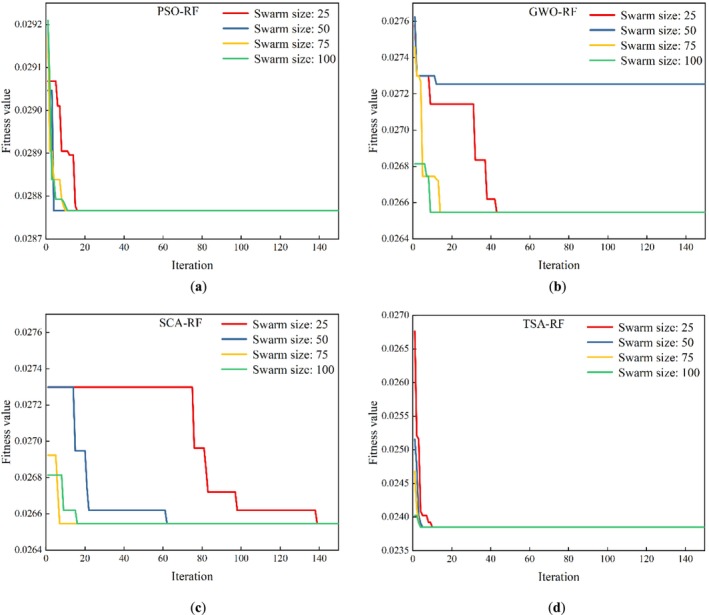
Optimization process of (a) PSO‐RF, (b) GWO‐RF, (c) SCA‐RF, and (d) TSA‐RF models.

To strike a balance between the accuracy and computational efficiency of the RF‐based hybrid models, the optimal swarm size and number of iterations were determined through multiple model constructions. The swarm sizes selected for each metaheuristic algorithm were 25, 50, 75, and 100, with the number of iterations fixed at 150. Figure 13 shows the optimization process of PSO‐RF, GWO‐RF, SCA‐RF, and TSA‐RF models. As can be seen from Figure 13a, all the computational results of the PSO‐RF model converge to the same value under the four swarm sizes (25, 50, 75, and 100), i.e., the final fitness values all converge to 0.028766. In this case, the swarm size that exhibits the fastest convergence is selected as the optimal swarm size. This ensures that the model enhances computational efficiency without compromising accuracy. From the figure, it can be seen that the PSO‐RF model exhibits the fastest convergence speed when the swarm size is 50. Therefore, the PSO‐RF model with a swarm size of 50 was selected as the best model among these four swarm sizes. From Figure 13b, it can be seen that the GWO‐RF model converged to the minimum fitness value (0.026547) for swarm sizes 25, 75, and 100. The accuracy of the model with these three swarm sizes was higher than that of the model with a swarm size of 50. Among these three models with different swarm sizes, the model with a swarm of 100 converged the fastest. Therefore, the model with a swarm size of 100 is the best model for GWO‐RF. As can be seen from Figure 13c, the SCA‐RF model converged to 0.026547 for all calculations with four swarm sizes, which matches the minimum fitness value required for the GWO‐RF model to converge. The swarm size that showed the fastest convergence was 75. Therefore, the SCA‐RF model with a swarm size of 75 was selected for the subsequent model evaluation. As can be seen in Figure 13d, all swarm model calculations for the TSA‐RF model converged to the same value (0.023849). The model showed the fastest convergence when the swarm size was 75. Therefore, this swarm size is considered the best model for TSA‐RF in all four cases.

From Figure 13, it can be seen that the minimum fitness value of the TSA‐RF model is smaller than the minimum fitness values of the PSO‐RF, GWO‐RF, and SCA‐RF models. However, further evaluation is required to determine the best RF‐based metaheuristic hybrid model. In what follows, a comparative analysis of five models (single RF model, PSO‐RF model with 50 swarms, GWO‐RF model with 100 swarms, SCA‐RF model with 75 swarms, and TSA‐RF model with 75 swarms) is presented.

**FIGURE 14:**
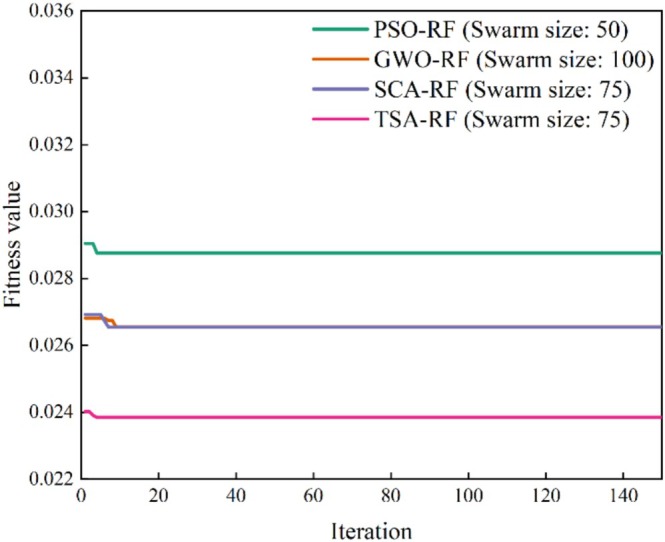
Comparison of optimal optimization processes for four hybrid models.

Figure 14 illustrates the comparison of the optimization processes for the four hybrid models. The optimization process of the best model for each method is as follows: the PSO‐RF model with 50 populations, the GWO‐RF model with 100 populations, the SCA‐RF model with 75 populations, and the TSA‐RF model with 75 populations. All four models demonstrate faster convergence, indicating their superior learning capabilities. Additionally, it is evident that the final fitness value (MSE) of the TSA‐RF model is lower than that of the PSO‐RF, GWO‐RF, and SCA‐RF models; however, the differences in the final fitness values among the four models are relatively minor. Therefore, further evaluation is necessary to determine the optimal RF‐based hybrid model.

Table 4 summarizes the final hyperparameters obtained for each optimization algorithm, along with the optimal parameter settings for each. It is important to note that these results were derived from the training dataset. The subsequent section of this study will discuss the specific performance of all models on both the training and test sets, ultimately selecting the best model for predicting grain porosity.

**TABLE 4:** Optimal parameters for tuning RF hyperparameters and setting metaheuristic algorithms.

Hybrid model	Hyperparameters		Optimal parameterization of metaheuristic algorithms	
	*ntree*	*mtry*	Number of iterations	Swim size
PSO‐RF	118	7	150	50
GWO‐RF	44	7	150	100
SCA‐RF	44	7	150	75
TSA‐RF	90	7	150	75

### 5.3 Selecting the Optimal Predictive Model

#### 5.3.1 Error Analysis

**FIGURE 15:**
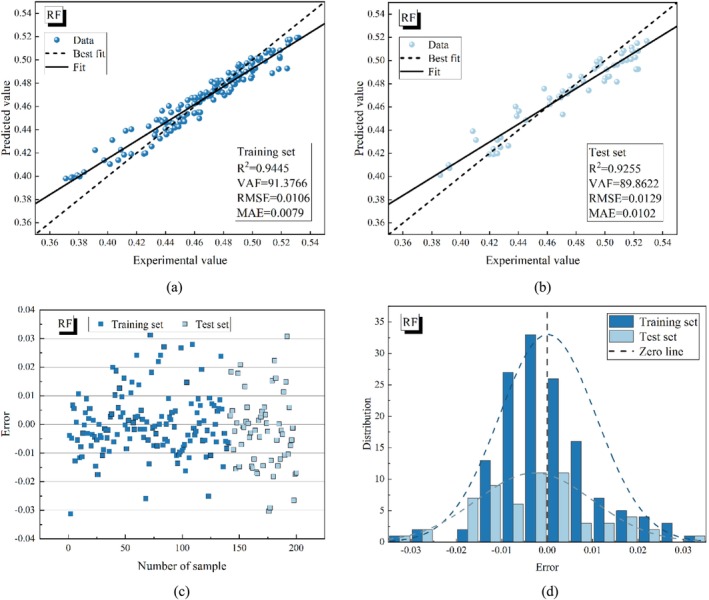
Porosity prediction results of the RF model (a) scatter plot of predicted and experimental values based on the training set, (b) scatter plot of predicted and experimental values based on the test set, (c) error scatter plot, and (d) error frequency histograms.

**FIGURE 16:**
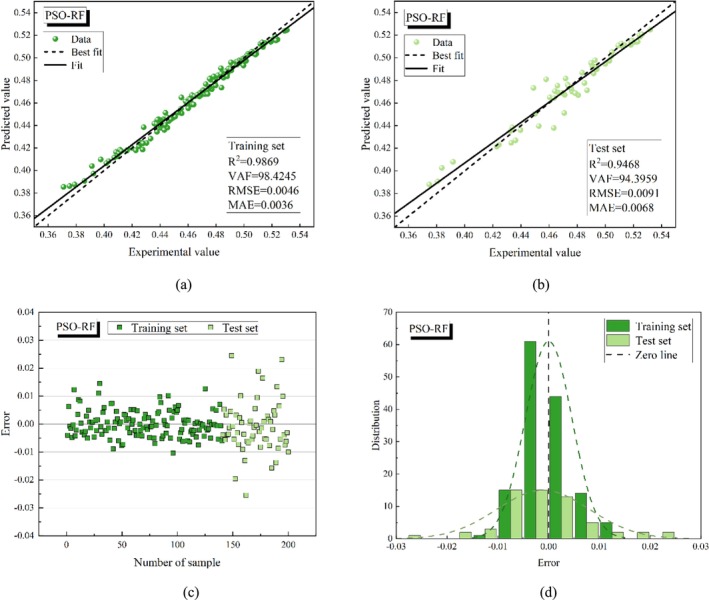
Porosity prediction results of the PSO‐RF model (a) scatter plot of predicted and experimental values based on the training set, (b) scatter plot of predicted and experimental values based on the test set, (c) error scatter plot, and (d) error frequency histograms.

**FIGURE 17:**
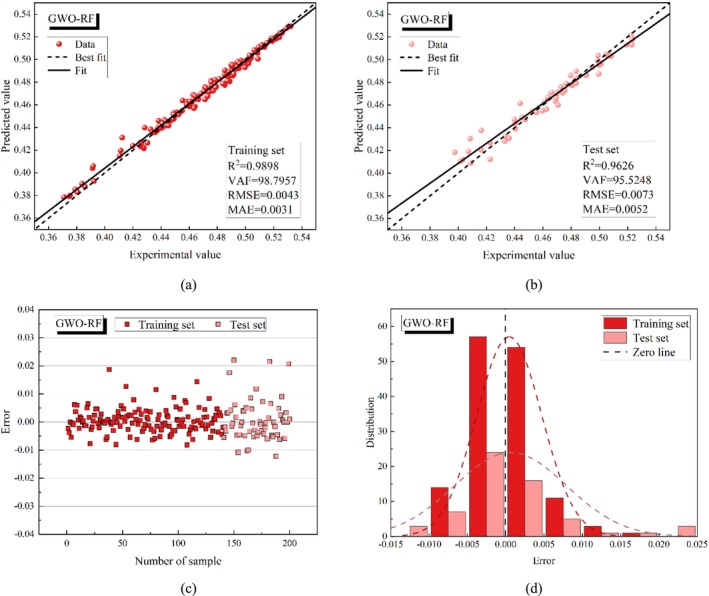
Porosity prediction results of the GWO‐RF model (a) scatter plot of predicted and experimental values based on the training set, (b) scatter plot of predicted and experimental values based on the test set, (c) error scatter plot, and (d) error frequency histograms.

**FIGURE 18:**
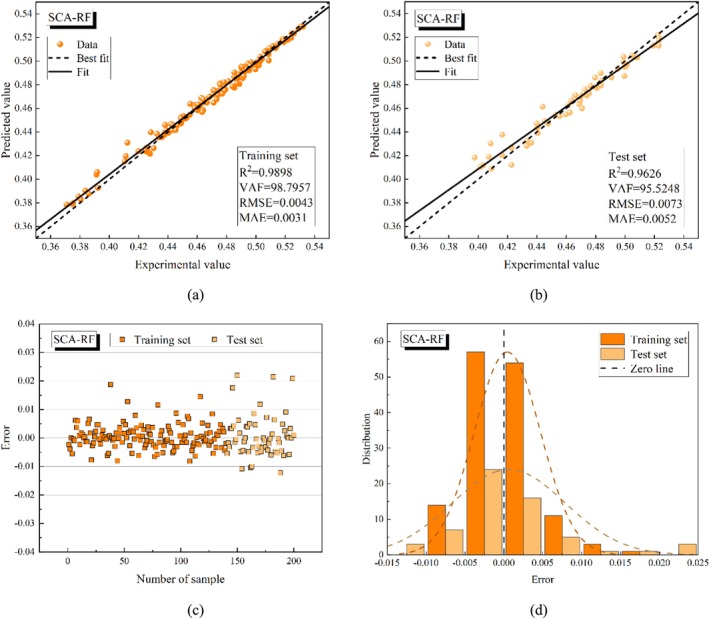
Porosity prediction results of the SCA‐RF model (a) scatter plot of predicted and experimental values based on the training set, (b) scatter plot of predicted and experimental values based on the test set, (c) error scatter plot, and (d) error frequency histograms.

**FIGURE 19:**
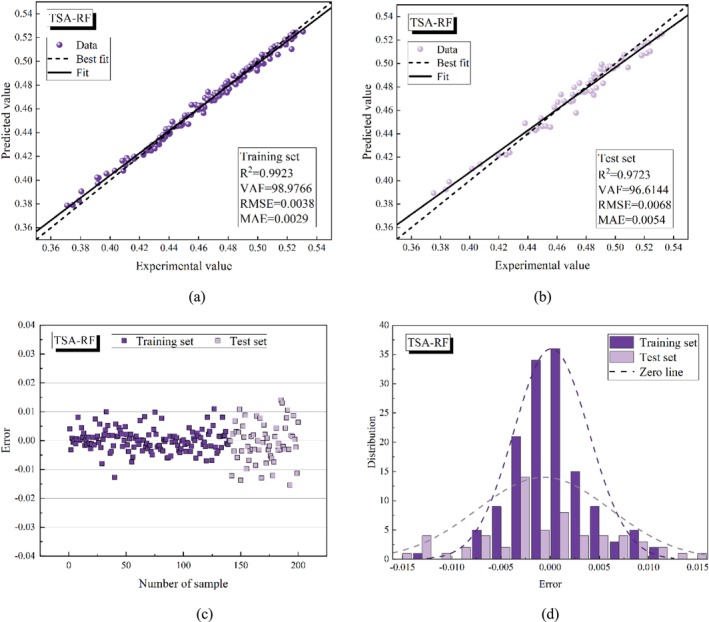
Porosity prediction results of the TSA‐RF model (a) scatter plot of predicted and experimental values based on the training set, (b) scatter plot of predicted and experimental values based on the test set, (c) error scatter plot, and (d) error frequency histograms.

Figures 15, 16, 17, 18, 19 present the porosity prediction results for the RF, PSO‐RF, GWO‐RF, SCA‐RF, and TSA‐RF models. Figures 15a,b through 19a,b display scatter plots comparing the predicted values of grain porosity against the experimental values for both the training and test sets, illustrating the correlation between the two. Additionally, Figures 15c,d through 19c,d depict scatter plots of error distributions alongside error frequency histograms. A distribution of errors closer to zero indicates higher model accuracy. The figures clearly show that the distribution of the predicted and experimental values of grain porosity for the TSA‐RF model, both in the training and test sets, is primarily concentrated around the best‐fit line. Furthermore, the error scatter of the TSA‐RF model is predominantly located near the zero point, and its distribution curve follows a Gaussian bell shape. This indicates that the grain porosity prediction results of the TSA‐RF model are superior to those of the other four models.

#### 5.3.2 Taylor Diagram

The Taylor diagram serves as a visual tool for evaluating how well a set of data aligns with observed values (Medawela et al. 2023). It illustrates the relationship between predicted and observed values in terms of the correlation coefficient, root mean square error, and standard deviation (Taylor 2005). In this study, Taylor diagrams were employed to visualize the discrepancies between the predicted and actual results of grain porosity from various prediction models (RF, PSO‐RF, GWO‐RF, SCA‐RF, and TSA‐RF). In the Taylor diagram, each model is represented by a point. It is important to note that, for an ideal model, the location of this point should coincide with the observed point.

**FIGURE 20:**
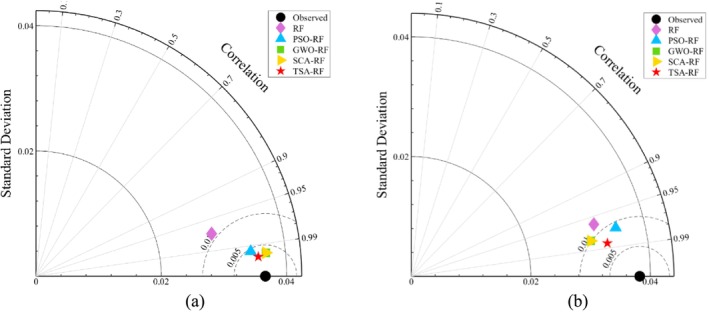
Taylor diagram (a) training set, and (b) test set.

Figure 20 illustrates the Taylor diagram comparing the prediction results from the RF and its hybrid models with the experimental results from both the training and test sets. The diagram indicates that the correlation coefficients for the RF models in the training set range from 0.95 to 0.99, while the correlation coefficients for the PSO‐RF, GWO‐RF, SCA‐RF, and TSA‐RF models exceed 0.99. In the test set, the correlation coefficients for RF, PSO‐RF, GWO‐RF, SCA‐RF, and TSA‐RF range from 0.95 to 0.99. Additionally, in both the training and test sets, the TSA‐RF model is the closest to the observed values. This indicates that the TSA‐RF model demonstrates higher predictive accuracy.

#### 5.3.3 Assessing Model Performance Using Evaluation Metrics

The four evaluation metrics of the five porosity prediction models were calculated according to Equations (29) through (32). Table 5 displays the performance evaluation metric values of the five ML models in both the training and test sets. As can be seen from the table, the results of all the performance evaluation metrics are very close. To select the best prediction model, a method proposed by Zorlu et al. (2008), which assigns a score or ranking to a model based on its predictive ability, was used. In other words, the higher the score or ranking of a model, the better its performance. Table 5 presents the scores of each model, including their performance on both the training and test sets. Based on the scores of each model, it can be concluded that the four hybrid models (PSO‐RF, GWO‐RF, SCA‐RF, and TSA‐RF) perform better on the training and test sets compared to the single RF model. Among the four hybrid models, the TSA‐RF model demonstrates superior performance and robustness on both the training and test sets, while PSO‐RF performs the worst in comparison.

**TABLE 5:** Porosity prediction performance of five ML models.

	Model	*R* ^2^	Score	VAF (%)	Score	RMSE	Score	MAE	Score	Score summation
Training set	RF	0.9445	2	91.3766	2	0.0106	2	0.0079	2	8
	PSO‐RF	0.9869	3	98.4245	3	0.0046	3	0.0036	3	12
	GWO‐RF	0.9898	4	98.7957	4	0.0043	4	0.0031	4	16
	SCA‐RF	0.9898	4	98.7957	4	0.0043	4	0.0031	4	16
	TSA‐RF	0.9923	5	98.9766	5	0.0038	5	0.0029	5	20
Test set	RF	0.9255	2	89.8622	2	0.0129	2	0.0102	2	8
	PSO‐RF	0.9468	3	94.3959	3	0.0091	3	0.0068	3	12
	GWO‐RF	0.9626	4	95.5248	4	0.0073	4	0.0052	5	17
	SCA‐RF	0.9626	4	95.5248	4	0.0073	4	0.0052	5	17
	TSA‐RF	0.9723	5	96.6144	5	0.0068	5	0.0054	4	19

**FIGURE 21:**
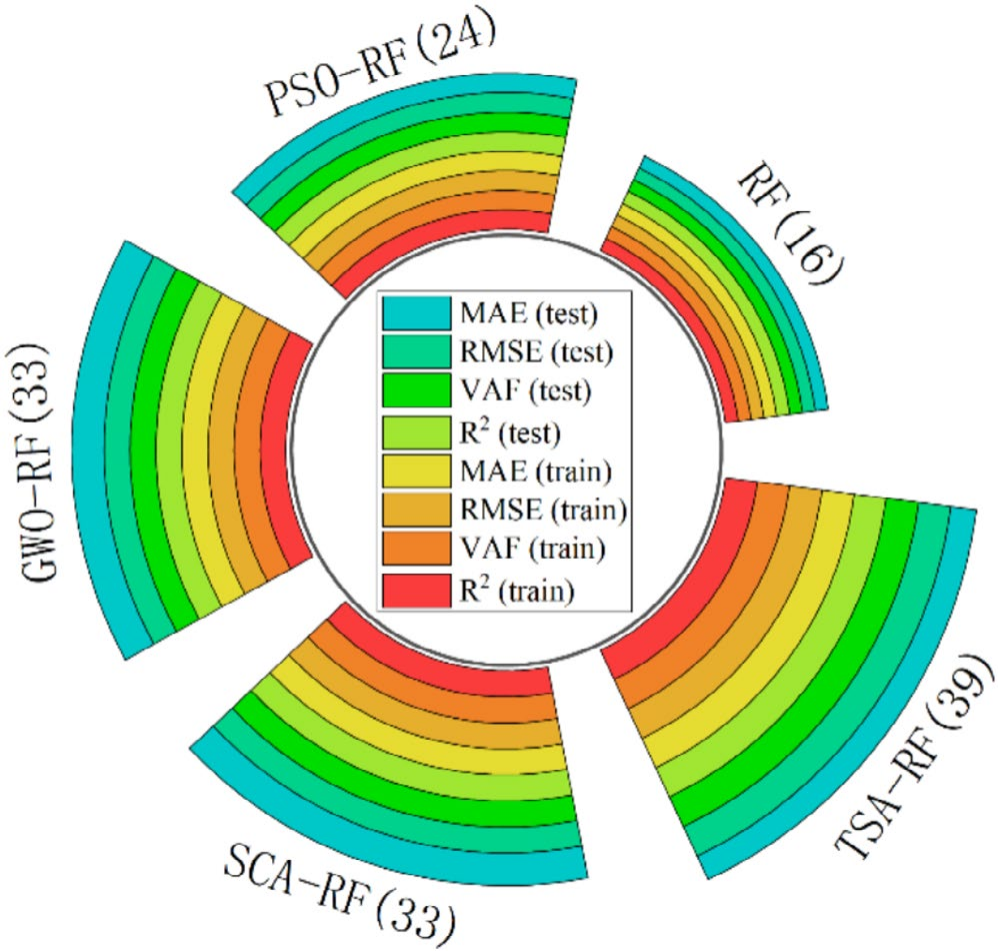
Total scores of RF, PSO‐RF, GWO‐RF, SCA‐RF, and TSA‐RF models for predicting porosity.

Figure 21 displays the total scores of the RF, PSO‐RF, GWO‐RF, SCA‐RF, and TSA‐RF models in predicting porosity. As can be seen in Figure 21, the TSA‐RF model performs the best among the five models, achieving a total score of 39. Notably, the combined score of GWO‐RF and SCA‐RF is 33, suggesting that the predictive accuracy of these two models is slightly inferior to that of the TSA‐RF model. The predictive performance of PSO‐RF is lower than that of GWO‐RF and SCA‐RF, with a total score of 24. All four RF‐based hybrid models outperform the single RF model, which scored 16, indicating that the metaheuristic optimization algorithm significantly enhances the prediction performance of the RF model. For example, by using the TSA‐RF hybrid model, the RMSE value of the training set can be reduced from 0.0106 to 0.0038, and the RMSE value of the test set can be reduced from 0.0129 to 0.0068. Therefore, in this study, the hyperparameters obtained from the TSA‐RF model are selected for further analysis.

#### 5.3.4 Multi‐Criteria Assessment

Although the evaluation criteria mentioned above are widely recognized statistical tools for assessing the accuracy of ML models, these metrics are susceptible to a degree of local linearity and bias. The study conducted by Gandomi et al. (2013) suggests that AI models should be validated through a rigorous multi‐criteria process that considers both traditional and modern performance metrics, as well as error matrices. This approach is preferable to assessing a model's accuracy based on a single parameter. Consequently, this study also employed the relative percentage difference (RPD) metric and the objective function (OBJ) to evaluate the performance of the five models. The formulas for RPD and OBJ are provided below:

(33) $$\mathrm{RPD}=\frac{\mathrm{SD}}{\text{RMSE}}$$

(34) $$\begin{array}{cc} \mathrm{OBJ}= & \left(\frac{m_{1}-m_{2}}{m_{1}+m_{2}}\right)\times \frac{{\text{RMSE}}_{1}+{\mathrm{MAE}}_{1}}{R_{1}^{2}+1}\\ & +\left(\frac{2m_{2}}{m_{1}+m_{2}}\right)\times \frac{{\text{RMSE}}_{2}+{\mathrm{MAE}}_{2}}{R_{2}^{2}+1} \end{array}$$

where SD represents the standard deviation of the measured observations, *m*_1_ denotes the number of samples in the training set, and *m*_2_ indicates the number of samples in the test set. RMSE_1_, MAE_1_, and $R_{1}^{2}$ refer to the RMSE, MAE, and $R^{2}$ values for the training set, while RMSE_2_, MAE_2_, and $R_{2}^{2}$ correspond to the RMSE, MAE, and $R^{2}$ values for the test set. When the RPD value is less than 1.4, it indicates that the model's predictions are extremely poor. An RPD value between 1.4 and 1.8 suggests that the model's predictions are marginal. If the RPD value falls between 1.8 and 2.0, it signifies fair predictions. An RPD value ranging from 2.0 to 2.5 indicates that the model predicts well. Finally, when the RPD value exceeds 2.5, it reflects excellent predictive performance (Rossel et al. 2006). The smaller the OBJ value, the greater the accuracy of the model.

**FIGURE 22:**
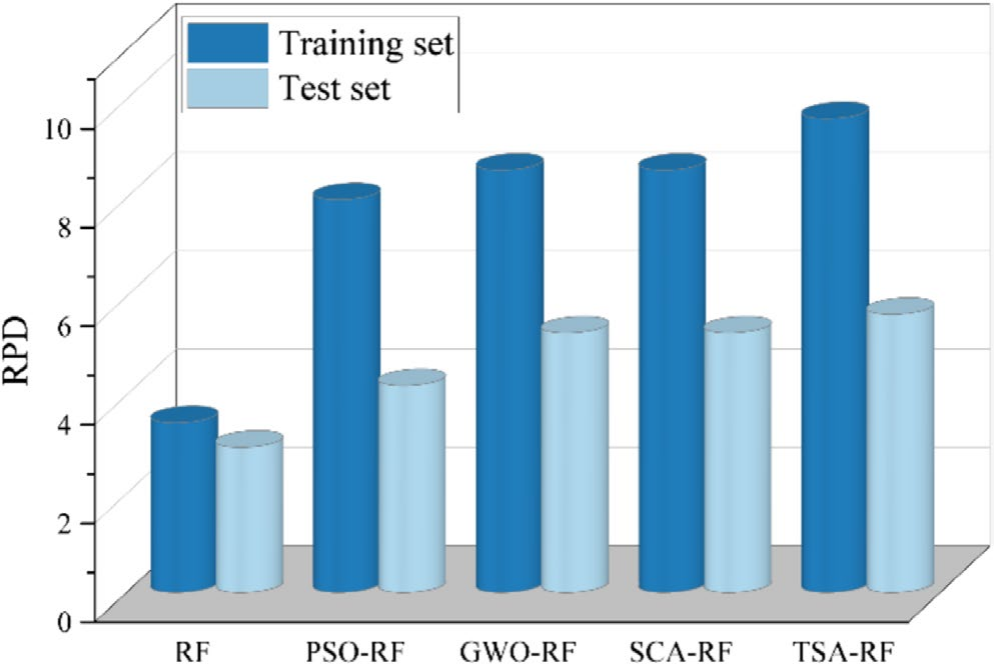
Model evaluation based on RPD metrics.

**FIGURE 23:**
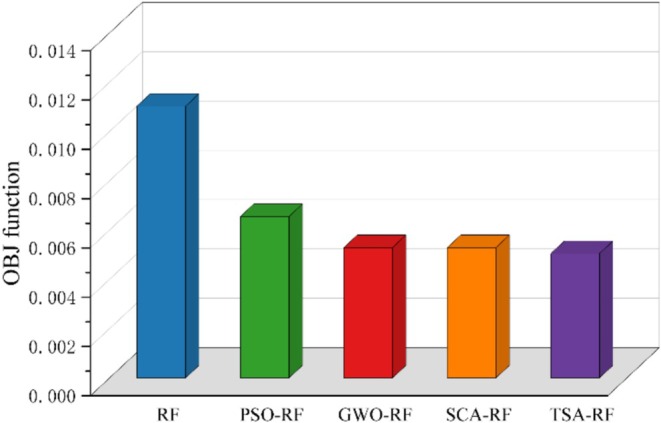
Model evaluation based on OBJ function.

Figure 22 illustrates the model evaluation based on RPD metrics. The data indicate that the RPD values for all five models in both the training and test sets exceed 2.5, suggesting that all models demonstrate strong performance in predicting grain porosity. Notably, the TSA‐RF model exhibits the highest RPD values in both the training set and the test set, with values of 9.6094 and 5.6396, respectively. This indicates that the TSA‐RF model possesses the most robust predictive capability. Figure 23 presents the model evaluation based on the OBJ function. The OBJ function values reflect the overall performance of the model in both the training and test sets. Lower values indicate higher accuracy, while higher values suggest lower accuracy. As shown in the figure, the TSA‐RF model has the lowest OBJ value, recorded at 0.005047.

## 6 TSA‐RF Model Applied to Predict the Porosity of Bulk Grain Piles in the Bungalow Warehouse

### 6.1 Bungalow Warehouse Experimental Setup

In this study, a bungalow warehouse in Henan Province, China, with geometric dimensions of 5.522 m (L) × 8.214 m (W) × 11 m (H), was used as the research object. The storage material in the bungalow warehouse was North China wheat, with an initial moisture content of 10.89%, a bulk density of 818.16 kg/m^3^, an internal friction angle of 30.114°, an internal friction coefficient of 0.58, and an external friction coefficient of 0.4. The grain loading method in the bungalow warehouse involved layered loading. The entire loading process was divided into 6 stages, with cumulative stacking heights of 0.40 m, 0.95 m, 1.80 m, 2.83 m, 4.04 m, and 5.00 m, respectively.

**FIGURE 24:**
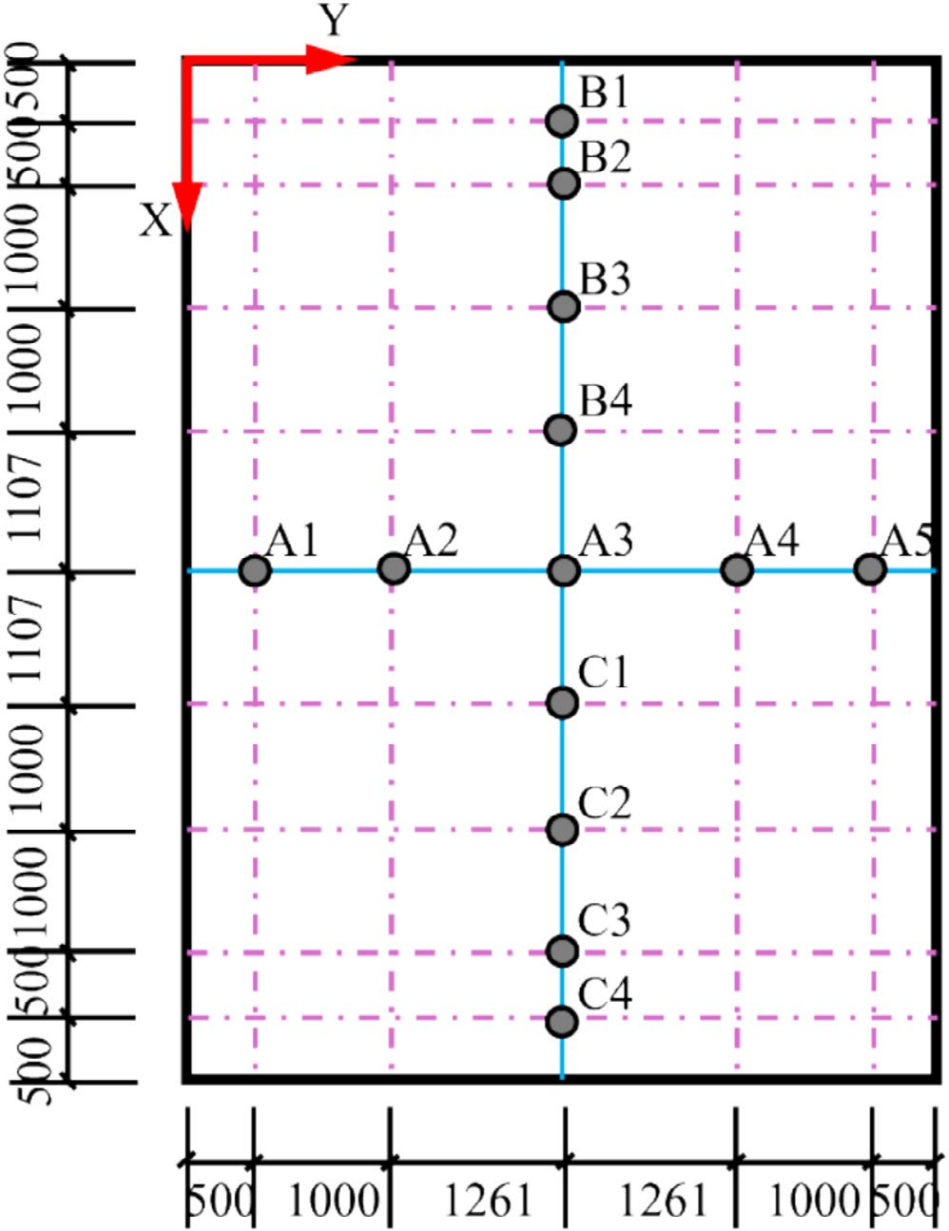
Distribution of pressure sensors at the bottom of the grain pile in the bungalow warehouse.

In order to determine the spatial distribution pattern of grain pile porosity in the bungalow warehouse, a total of nine monitoring points were positioned along the length direction of the warehouse, namely B1, B2, B3, B4, A3, C1, C2, C3, and C4. Additionally, five monitoring points were placed along the width direction of the warehouse, namely A1, A2, A3, A4, and A5. The specific distribution of each monitoring point in the bungalow warehouse is illustrated in Figure 24, with the long side of the warehouse designated as the X‐axis direction and the short side as the Y‐axis direction. During the experiment, the pressure at the bottom of the warehouse was recorded at various stacking heights. Tables 6 and 7 display the pressure values at different stacking heights in the bungalow warehouse at mid‐plumb Y = 2.761 m and mid‐plumb X = 4.107 m, respectively.

**TABLE 6:** Pressure values at each measurement point at various stacking heights in the bungalow warehouse (mid‐plumb Y = 2.761 m).

Stacking height/m	Monitoring point B1	Monitoring point B2	Monitoring point B3	Monitoring point B4	Monitoring point A3	Monitoring point C1	Monitoring point C2	Monitoring point C3	Monitoring point C4
0.40	0.446	0.372	1.933	2.082	1.264	0.595	1.561	0.372	0.370
0.95	3.284	3.582	5.821	6.194	5.149	5.373	5.299	3.955	3.507
1.80	9.212	9.723	10.968	12.288	11.970	10.685	10.964	10.128	9.375
2.83	15.419	16.337	18.173	19.636	20.539	17.168	17.330	17.400	15.623
4.04	21.859	23.941	25.112	27.584	29.535	25.892	23.941	24.981	22.770
5.00	23.922	26.264	30.279	33.123	34.293	31.115	28.606	27.435	25.260

**TABLE 7:** Pressure values at each measurement point at various stacking heights in the bungalow warehouse (mid‐plumb X = 4.107 m).

Stacking height/m	Monitoring point A1	Monitoring point A2	Monitoring point A3	Monitoring point A4	Monitoring point A5
0.40	1.963	1.986	1.264	1.594	1.538
0.95	5.126	5.207	5.149	4.983	4.847
1.80	9.212	10.723	11.970	11.288	10.670
2.83	16.853	18.372	20.539	17.536	16.939
4.04	24.923	26.891	29.535	27.374	25.247
5.00	28.752	31.167	34.293	32.026	29.173

### 6.2 Prediction of Bulk Grain Pile Porosity in the Bungalow Warehouse Based on the TSA‐RF Model

**FIGURE 25:**
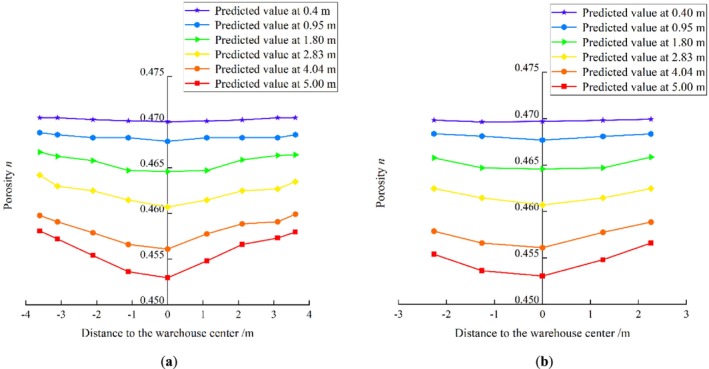
Bulk wheat prediction results from the TSA‐RF model for the bungalow warehouse experiment (a) mid‐plumb Y = 2.761 m, and (b) mid‐plumb X = 4.107 m.

The results of the bungalow warehouse experiments were incorporated into the constructed TSA‐RF model to predict bulk wheat porosity in a hierarchical manner. Figure 25 shows the TSA‐RF model's predictions for bulk wheat porosity in the bungalow warehouse experiment. Figure 25 indicates that the grain pile porosity in both the X‐axis and Y‐axis directions exhibited an axisymmetric distribution. Additionally, from a vertical perspective, the wheat pile porosity displayed a stratified phenomenon. In the central vertical plane, the mean porosity values at the top and bottom of the grain pile were 0.470 and 0.455 in the X‐ and Y‐axis directions, respectively. When the depth of the wheat pile was between 0.4 and 0.95 m, the average porosity decreased at a rate of 0.382%. When the depth was between 4.04 and 5 m, the average porosity decreased at a rate of 0.128%. The porosity of the top layer remained stable with increasing distance from the centerline of the bungalow warehouse. The porosity of the bottom layer exhibited a trend of ‘small in the middle and large around’ with increasing distance from the centerline. The trend of porosity change became more pronounced closer to the bottom layer of the grain pile. The main reason for this phenomenon is that pressure increases closer to the bottom layer of the grain pile, particularly near the center of the warehouse. Additionally, friction from the warehouse wall affects the grain pile near the edges. As a result, the porosity in the middle of the bottom layer is smaller, while the porosity in the surrounding area is larger.

**FIGURE 26:**
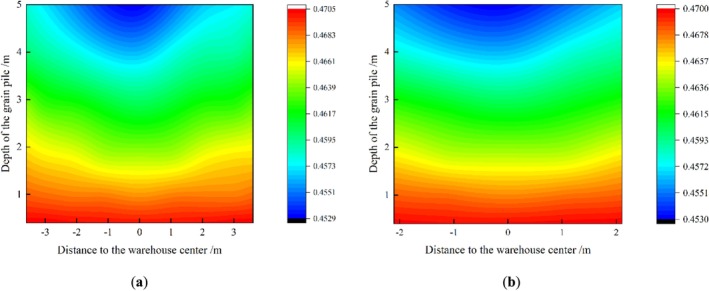
Clouds of porosity distribution at different depths within the wheat grain pile (a) mid‐plumb Y = 2.761 m, and (b) mid‐plumb X = 4.107 m.

Figure 26 shows the porosity cloud at different depths inside the wheat grain pile at mid‐plumb Y = 2.761 m and mid‐plumb X = 4.107 m. From the cloud diagram, it is evident that at the same depth in the bungalow warehouse, the porosity decreases toward the center of the warehouse and increases toward the wall. This trend becomes more pronounced as the depth of the grain pile increases. The porosity of the wheat grain pile increases with depth, exhibiting a stratified distribution. Near the bottom of the grain pile, the porosity shows a more pronounced concave stratification.

## 7 Limitations and Future Outlook

Although the proposed TSA‐RF hybrid model demonstrates high reliability and accuracy, this study has several limitations that warrant further investigation. First, due to constraints in the experimental setup, only 200 data points were obtained for the construction of the ML model. Since model performance heavily depends on the quantity and quality of training data, future research should expand the range of input variables and target parameters to establish a more comprehensive and higher‐quality database. Second, while this study considered seven key factors influencing grain porosity, it did not consider all potential variables. To improve the accuracy and reliability of the model, future experimental designs and data collection processes should incorporate a broader set of influencing factors. Finally, although the TSA algorithm effectively improved the predictive performance of the RF model, numerous other optimization algorithms remain unexplored, such as the Multiverse Optimization Algorithm, Whale Optimization Algorithm, and Harris Hawk Optimization Algorithm. Subsequent studies will investigate these and other optimization algorithms to further refine ML methodologies.

## 8 Conclusion

In this study, experimental data on grain porosity and its influencing factors were obtained through grain compression experiments. A series of analyses were conducted on the experimental data to establish a database for ML. Five ML models (RF, PSO‐RF, GWO‐RF, SCA‐RF, and TSA‐RF models) were constructed, and their predictive performance was compared using various evaluation methods to select the optimal model (TSA‐RF model). Finally, the TSA‐RF model was employed to predict the porosity of bulk wheat heaps in cottage silos in a hierarchical manner. The main conclusions of this study are as follows:

1. To evaluate the experimental data obtained from the grain compression experiments, a series of analyses were conducted. The results indicated that the data distribution of each variable was uniform, with no obvious outliers, making the experimental data suitable for use as a database for ML.
2. In model development, when the number of iterations was set to 150, the PSO‐RF model with a swarm size of 50, the GWO‐RF model with a swarm size of 100, the SCA‐RF model with a swarm size of 75, and the TSA‐RF model with a swarm size of 75 were identified as the most effective predictive models for the hybrid model, achieving a balance between accuracy and computational efficiency.
3. The prediction results of the five models were analyzed using error analysis, a Taylor diagram, four evaluation metrics, and a multi‐criteria assessment. The analysis revealed that all four RF‐based hybrid models outperformed the single RF model, with the TSA‐RF model exhibiting the best predictive performance among the hybrid models.
4. The TSA‐RF model was applied to predict the porosity of bulk wheat piles in a warehouse. The prediction results revealed that the porosity of the wheat grain pile at the same height in the bungalow warehouse exhibited an axisymmetric distribution. The porosity trend remained stable at each measurement point on the top layer of the grain pile. As the depth of the grain pile increases, the porosity within the bungalow warehouse displayed a pattern of “decreasing in the center and increasing around the edges.” Furthermore, the closer to the bottom layer of the grain pile, the more pronounced this porosity trend became. The porosity of the wheat grain pile increased with the depth of the grain pile, demonstrating a stratified distribution phenomenon. Near the bottom of the grain pile, the porosity exhibited a more pronounced concave stratification.

## Author Contributions

**Jiahao Chen:** conceptualization (equal), formal analysis (equal), methodology (equal), resources (equal), supervision (equal), writing – review and editing (equal). **Jiaxin Li:** conceptualization (equal), data curation (lead), formal analysis (equal), investigation (equal), methodology (equal), software (lead), validation (lead), writing – original draft (lead), writing – review and editing (equal). **Deqian Zheng:** project administration (equal), resources (equal), supervision (equal). **Yan Zhang:** investigation (equal), validation (supporting). **Hang Jing:** project administration (equal). **Jianjun Han:** writing – review and editing (supporting). **Manxing Wang:** validation (supporting). **Runmei Zhao:** data curation (supporting).

## Conflicts of Interest

The authors declare no conflicts of interest.

## Data Availability

The original contributions presented in the study are included in the article; further inquiries can be directed to the corresponding author.

## References

[fsn370107-bib-0001] Al Khalifah, H. , P. Glover , and P. Lorinczi . 2020. “Permeability Prediction and Diagenesis in Tight Carbonates Using Machine Learning Techniques.” Marine and Petroleum Geology 112: 104096. 10.1016/j.marpetgeo.2019.104096.

[fsn370107-bib-0002] Breiman, L. 2001. “Random Forests.” Machine Learning 45: 5–32. 10.1023/A:1010933404324.

[fsn370107-bib-0003] Coşkun, M. B. , İ. Yalçın , and C. Özarslan . 2006. “Physical Properties of Sweet Corn Seed (*Zea mays saccharata* Sturt.).” Journal of Food Engineering 74, no. 4: 523–528. 10.1016/j.jfoodeng.2005.03.039.

[fsn370107-bib-0004] Duan, S. , W. Yang , X. Wang , S. Mao , and Y. Zhang . 2019. “Forecasting of Grain Pile Temperature From Meteorological Factors Using Machine Learning.” IEEE Access 7: 130721–130733. 10.1109/ACCESS.2019.2940266.

[fsn370107-bib-0005] Eliopoulos, P. , I. Potamitis , D. C. Kontodimas , and E. Givropoulou . 2015. “Detection of Adult Beetles Inside the Stored Wheat Mass Based on Their Acoustic Emissions.” Journal of Economic Entomology 108, no. 6: 2808–2814. 10.1093/jee/tov231. 26470377

[fsn370107-bib-0006] Gandomi, A. H. , G. J. Yun , and A. H. Alavi . 2013. “An Evolutionary Approach for Modeling of Shear Strength of RC Deep Beams.” Materials and Structures 46: 2109–2119. 10.1617/s11527-013-0039-z.

[fsn370107-bib-0007] Ge, M. , G. Chen , C. Liu , D. Zheng , and W. Liu . 2023. “Study of the Pore Structure Characteristics of Soybean Grain Piles Using Image Processing Technology.” International Agrophysics 37, no. 2: 201–214. 10.31545/intagr/162959.

[fsn370107-bib-0008] Ghodki, B. M. , M. Patel , R. Namdeo , and G. Carpenter . 2019. “Calibration of Discrete Element Model Parameters: Soybeans.” Computational Particle Mechanics 6: 3–10. 10.1007/s40571-018-0194-7.

[fsn370107-bib-0009] Guo, Y. , D. Tang , W. Tang , et al. 2022. “Agricultural Price Prediction Based on Combined Forecasting Model Under Spatial–Temporal Influencing Factors.” Sustainability 14, no. 17: 10483. 10.3390/su141710483.

[fsn370107-bib-0010] Gustafson, R. J. , and G. E. Hall . 1972. “Density and Porosity Changes of Shelled Corn During Drying.” Transactions of ASAE 15, no. 3: 523–525. 10.13031/2013.37944.

[fsn370107-bib-0011] Han, D. , and X. Xue . 2024. “Machine Learning‐Based Prediction of Shear Strength Parameters of Rock Materials.” Rock Mechanics and Rock Engineering 57, no. 10: 1–25. 10.1007/s00603-024-04012-3.

[fsn370107-bib-0012] Han, Y. , D. Kwak , S. Q. Choi , C. Shin , Y. Lee , and H. Kim . 2017. “Pore Structure Characterization of Shale Using Gas Physisorption: Effect of Chemical Compositions.” Minerals 7, no. 5: 66. 10.3390/min7050066.

[fsn370107-bib-0013] He, B. , D. J. Armaghani , and S. H. Lai . 2023. “Assessment of Tunnel Blasting‐Induced Overbreak: A Novel Metaheuristic‐Based Random Forest Approach.” Tunnelling and Underground Space Technology 133: 104979. 10.1016/j.tust.2022.104979.

[fsn370107-bib-0014] Hou, K. , M. Guo , X. Li , and H. Zhang . 2021. “Research on Optimization of GWO‐BP Model for Cloud Server Load Prediction.” IEEE Access 9: 162581–162589. 10.1109/ACCESS.2021.3132052.

[fsn370107-bib-0015] Jayas, D. , N. White , and W. Muir . 1995. “Stored‐Grain Ecosystem.” Drying Technology 13, no. 4: 1045–1046. 10.1080/07373939508917007.

[fsn370107-bib-0016] Jia, Y. , Z. Li , R. Gao , X. Zhang , H. Zhang , and Z. Su . 2022. “Mildew Recognition on Maize Seed by Use of Hyperspectral Technology.” Spectroscopy Letters 55, no. 4: 240–249. 10.1080/00387010.2022.2053163.

[fsn370107-bib-0017] Kaur, S. , L. K. Awasthi , A. L. Sangal , and G. Dhiman . 2020. “Tunicate Swarm Algorithm: A New Bio‐Inspired Based Metaheuristic Paradigm for Global Optimization.” Engineering Applications of Artificial Intelligence 90: 103–541. 10.1016/j.engappai.2020.103541.

[fsn370107-bib-0018] Kennedy, J. , and R. Eberhart . 1995. “Particle Swarm Optimization.” Paper Presented at the Proceedings of ICNN'95‐International Conference on Neural Networks. 10.1109/ICNN.1995.488968.

[fsn370107-bib-0019] Koopialipoor, M. , D. Jahed Armaghani , M. Haghighi , and E. N. Ghaleini . 2019. “A Neuro‐Genetic Predictive Model to Approximate Overbreak Induced by Drilling and Blasting Operation in Tunnels.” Bulletin of Engineering Geology and the Environment 78: 981–990. 10.1007/s10064-017-1116-2.

[fsn370107-bib-0020] Lawrence, J. , D. E. Maier , and R. L. Stroshine . 2013. “Three‐Dimensional Transient Heat, Mass, Momentum, and Species Transfer in the Stored Grain Ecosystem: Part I. Model Development and Evaluation.” Transactions of the ASABE 56, no. 1: 179–188. 10.13031/2013.42569.

[fsn370107-bib-0021] Lei, Y. , S. Zhou , X. Luo , S. Niu , and N. Jiang . 2022. “A Comparative Study of Six Hybrid Prediction Models for Uniaxial Compressive Strength of Rock Based on Swarm Intelligence Optimization Algorithms.” Frontiers in Earth Science 10: 930130. 10.3389/feart.2022.930130.

[fsn370107-bib-0022] Li, G. , X. Ma , and H. Yang . 2018. “A Hybrid Model for Forecasting Sunspots Time Series Based on Variational Mode Decomposition and Backpropagation Neural Network Improved by Firefly Algorithm.” Computational Intelligence and Neuroscience 2018: 3713410. 10.1155/2018/3713410. 30405707 PMC6204195

[fsn370107-bib-0023] Liu, C. , G. Chen , Y. Zhou , D. Zheng , and Z. Zhang . 2022. “Element Tests and Simulation of Effects of Vertical Pressure on Compression and Mildew of Wheat.” Computers and Electronics in Agriculture 203: 107–447. 10.1016/j.compag.2022.107447.

[fsn370107-bib-0024] Lu, B. , F. Han , J. H. Aheto , M. M. Rashed , and Z. Pan . 2021. “Artificial Bionic Taste Sensors Coupled With Chemometrics for Rapid Detection of Beef Adulteration.” Food Science & Nutrition 9, no. 9: 5220–5228. 10.1002/fsn3.2494. 34532030 PMC8441491

[fsn370107-bib-0025] Mahmoodzadeh, A. , H. R. Nejati , M. Mohammadi , et al. 2022. “Prediction of Mode‐I Rock Fracture Toughness Using Support Vector Regression With Metaheuristic Optimization Algorithms.” Engineering Fracture Mechanics 264: 108334. 10.1016/j.engfracmech.2022.108334.

[fsn370107-bib-0026] Manandhar, A. , P. Milindi , and A. Shah . 2018. “An Overview of the Post‐Harvest Grain Storage Practices of Smallholder Farmers in Developing Countries.” Agriculture 8, no. 4: 57. 10.3390/agriculture8040057.

[fsn370107-bib-0027] Markussen, Ø. , H. Dypvik , E. Hammer , H. Long , and Ø. Hammer . 2019. “3D Characterization of Porosity and Authigenic Cementation in Triassic Conglomerates/Arenites in the Edvard Grieg Field Using 3D Micro‐CT Imaging.” Marine and Petroleum Geology 99: 265–281. 10.1016/j.marpetgeo.2018.10.015.

[fsn370107-bib-0028] Medawela, S. , D. J. Armaghani , B. Indraratna , R. K. Rowe , and N. Thamwattana . 2023. “Development of an Advanced Machine Learning Model to Predict the pH of Groundwater in Permeable Reactive Barriers (PRBs) Located in Acidic Terrain.” Computers and Geotechnics 161: 105557. 10.1016/j.compgeo.2023.105557.

[fsn370107-bib-0029] Meng, Q. , S. Feng , T. Tan , Q. Wen , and J. Shang . 2024. “Fast Detection of Moisture Content and Freshness for Loquats Using Optical Fiber Spectroscopy.” Food Science & Nutrition 12: 4819–4830. 10.1002/fsn3.4130. 39055228 PMC11266933

[fsn370107-bib-0030] Meng, Y. , S. Yu , H. Wang , J. Qin , and Y. Xie . 2019. “Data‐Driven Modeling Based on Kernel Extreme Learning Machine for Sugarcane Juice Clarification.” Food Science & Nutrition 7, no. 5: 1606–1614. 10.1002/fsn3.985. 31139373 PMC6526666

[fsn370107-bib-0031] Mirjalili, S. 2016. “SCA: A Sine Cosine Algorithm for Solving Optimization Problems.” Knowledge‐Based Systems 96: 120–133. 10.1016/j.knosys.2015.12.022.

[fsn370107-bib-0032] Mirjalili, S. , S. M. Mirjalili , and A. Lewis . 2014. “Grey Wolf Optimizer.” Advances in Engineering Software 69: 46–61. 10.1016/j.advengsoft.2013.12.007.

[fsn370107-bib-0033] Neethirajan, S. , and D. S. Jayas . 2008. “Analysis of Pore Network in Three‐Dimensional (3D) Grain Bulks Using X‐Ray CT Images.” Transport in Porous Media 73: 319–332. 10.1007/s11242-007-9172-x.

[fsn370107-bib-0034] Olatunde, G. , G. G. Atungulu , and S. Sadaka . 2016. “CFD Modeling of Air Flow Distribution in Rice Bin Storage System With Different Grain Mass Configurations.” Biosystems Engineering 151: 286–297. 10.1016/j.biosystemseng.2016.09.007.

[fsn370107-bib-0035] Raja, M. N. A. , S. T. A. Jaffar , A. Bardhan , and S. K. Shukla . 2023. “Predicting and Validating the Load‐Settlement Behavior of Large‐Scale Geosynthetic‐Reinforced Soil Abutments Using Hybrid Intelligent Modeling.” Journal of Rock Mechanics and Geotechnical Engineering 15, no. 3: 773–788. 10.1016/j.jrmge.2022.04.012.

[fsn370107-bib-0036] Rossel, R. V. , R. McGlynn , and A. McBratney . 2006. “Determining the Composition of Mineral‐Organic Mixes Using UV–Vis–NIR Diffuse Reflectance Spectroscopy.” Geoderma 137, no. 1–2: 70–82. 10.1016/j.geoderma.2006.07.004.

[fsn370107-bib-0037] Saki, M. , S. Siahpoush , A. R. Khaz'ali , and P. Technology . 2020. “A New Generalized Equation for Estimation of Sandstone and Carbonate Permeability From Mercury Intrusion Porosimetry Data.” Journal of Petroleum Exploration and Production Technology 10, no. 7: 2637–2644. 10.1007/s13202-020-00900-w.

[fsn370107-bib-0038] Salehi, H. , and R. Burgueño . 2018. “Emerging Artificial Intelligence Methods in Structural Engineering.” Engineering Structures 171: 170–189. 10.1016/j.engstruct.2018.05.084.

[fsn370107-bib-0039] Santiago, R. M. C. , S. L. Rabano , R. K. D. Billones , E. J. Calilung , E. Sybingco , and E. P. Dadios . 2017. “Insect Detection and Monitoring in Stored Grains Using MFCCs and Artificial Neural Network.” Paper Presented at the TENCON 2017–2017 IEEE Region 10 Conference. 10.1109/TENCON.2017.8228290.

[fsn370107-bib-0040] Shen, Y. , H. Zhou , J. Li , F. Jian , and D. S. Jayas . 2018. “Detection of Stored‐Grain Insects Using Deep Learning.” Computers and Electronics in Agriculture 145: 319–325. 10.1016/j.compag.2017.11.039.

[fsn370107-bib-0041] Shi, Z. , H. Dang , Z. Liu , and X. Zhou . 2020. “Detection and Identification of Stored‐Grain Insects Using Deep Learning: A More Effective Neural Network.” IEEE Access 8: 163703–163714. 10.1109/ACCESS.2020.3021830.

[fsn370107-bib-0042] Singh, S. P. , and S. C. Sharma . 2018. “A PSO Based Improved Localization Algorithm for Wireless Sensor Network.” Wireless Personal Communications 98: 487–503. 10.1007/s11277-017-4880-1.

[fsn370107-bib-0043] Sobieski, W. , Q. Zhang , and C. Liu . 2012. “Predicting Tortuosity for Airflow Through Porous Beds Consisting of Randomly Packed Spherical Particles.” Transport in Porous Media 93: 431–451. 10.1007/s11242-012-9961-8.

[fsn370107-bib-0044] Soulaine, C. , F. Gjetvaj , C. Garing , et al. 2016. “The Impact of Sub‐Resolution Porosity of X‐Ray Microtomography Images on the Permeability.” Transport in Porous Media 113: 227–243. 10.1007/s11242-016-0690-2.

[fsn370107-bib-0045] Sun, H. , H. V. Burton , and H. Huang . 2021. “Machine Learning Applications for Building Structural Design and Performance Assessment: State‐of‐the‐Art Review.” Journal of Building Engineering 33: 101816. 10.1016/j.jobe.2020.101816.

[fsn370107-bib-0046] Sun, Y. , J. Zhang , Z. Yu , Z. Liu , and P. Yin . 2022. “WOA (Whale Optimization Algorithm) Optimizes Elman Neural Network Model to Predict Porosity Value in Well Logging Curve.” Energies 15, no. 12: 4456. 10.3390/en15124456.

[fsn370107-bib-0047] Tanveer, M. U. , K. Munir , A. Raza , et al. 2025. “Novel Transfer Learning Approach for Detecting Infected and Healthy Maize Crop Using Leaf Images.” Food Science & Nutrition 13, no. 1: e4655. 10.1002/fsn3.4655. 39803246 PMC11717004

[fsn370107-bib-0048] Taylor, K. E. 2005. “Taylor Diagram Primer.” *Working Paper*, 1–4.

[fsn370107-bib-0049] Tongal, H. , and M. J. Booij . 2018. “Simulation and Forecasting of Streamflows Using Machine Learning Models Coupled With Base Flow Separation.” Journal of Hydrology 564: 266–282. 10.1016/j.jhydrol.2018.07.004.

[fsn370107-bib-0050] Wang, S. , K. Guan , C. Zhang , et al. 2022. “Using Soil Library Hyperspectral Reflectance and Machine Learning to Predict Soil Organic Carbon: Assessing Potential of Airborne and Spaceborne Optical Soil Sensing.” Remote Sensing of Environment 271: 112914. 10.1016/j.rse.2022.112914.

[fsn370107-bib-0051] Wu, A. , J. Zhu , Y. Yang , et al. 2018. “Classification of Corn Kernels Grades Using Image Analysis and Support Vector Machine.” Advances in Mechanical Engineering 10, no. 12: 1687814018817642. 10.1177/1687814018817642.

[fsn370107-bib-0052] Yaseen, Z. M. 2021. “An Insight Into Machine Learning Models Era in Simulating Soil, Water Bodies and Adsorption Heavy Metals: Review, Challenges and Solutions.” Chemosphere 277: 130126. 10.1016/j.chemosphere.2021.130126. 33774235

[fsn370107-bib-0053] Yu, Z. , X. Shi , J. Zhou , X. Chen , and X. Qiu . 2020. “Effective Assessment of Blast‐Induced Ground Vibration Using an Optimized Random Forest Model Based on a Harris Hawks Optimization Algorithm.” Applied Sciences 10, no. 4: 1403. 10.3390/app10041403.

[fsn370107-bib-0054] Yue, R. , and Q. Zhang . 2017. “A Pore‐Scale Model for Predicting Resistance to Airflow in Bulk Grain.” Biosystems Engineering 155: 142–151. 10.1016/j.biosystemseng.2016.12.007.

[fsn370107-bib-0055] Yuzgec, U. , Y. Becerikli , and M. Turker . 2006. “Nonlinear Predictive Control of a Drying Process Using Genetic Algorithms.” ISA Transactions 45, no. 4: 589–602. 10.1016/S0019-0578(07)60234-1. 17063940

[fsn370107-bib-0056] Zaheer, K. , S. Saeed , and S. Tariq . 2023. “Prediction of Aerosol Optical Depth Over Pakistan Using Novel Hybrid Machine Learning Model.” Acta Geophysica 71, no. 4: 2009–2029. 10.1007/s11600-023-01072-x.

[fsn370107-bib-0057] Zhou, J. , P. Yang , C. Li , and K. Du . 2023. “Hybrid Random Forest‐Based Models for Predicting Shear Strength of Structural Surfaces Based on Surface Morphology Parameters and Metaheuristic Algorithms.” Construction and Building Materials 409: 133911. 10.1016/j.conbuildmat.2023.133911.

[fsn370107-bib-0058] Zorlu, K. , C. Gokceoglu , F. Ocakoglu , H. Nefeslioglu , and S. Acikalin . 2008. “Prediction of Uniaxial Compressive Strength of Sandstones Using Petrography‐Based Models.” Engineering Geology 96, no. 3–4: 141–158. 10.1016/j.enggeo.2007.10.009.

